# Amino acid metabolism in immune cells: essential regulators of the effector functions, and promising opportunities to enhance cancer immunotherapy

**DOI:** 10.1186/s13045-023-01453-1

**Published:** 2023-06-05

**Authors:** Luming Yang, Zhaole Chu, Meng Liu, Qiang Zou, Jinyang Li, Qin Liu, Yazhou Wang, Tao Wang, Junyu Xiang, Bin Wang

**Affiliations:** 1 Chongqing University Medical School, Chongqing, 400044 People’s Republic of China; 2 Department of Gastroenterology and Chongqing Key Laboratory of Digestive Malignancies, Daping Hospital, Army Medical University (Third Military Medical University), 10# Changjiang Branch Road, Yuzhong District, Chongqing, 400042 People’s Republic of China; 3 Institute of Pathology and Southwest Cancer Center, Key Laboratory of Tumor Immunopathology of Ministry of Education of China, Southwest Hospital, Army Medical University (Third Military Medical University), Chongqing, 400038 People’s Republic of China; 4 Jinfeng Laboratory, Chongqing, 401329 People’s Republic of China

**Keywords:** Tumor microenvironment, Immune cells, Amino acids, SLC transporters, mTOR

## Abstract

Amino acids are basic nutrients for immune cells during organ development, tissue homeostasis, and the immune response. Regarding metabolic reprogramming in the tumor microenvironment, dysregulation of amino acid consumption in immune cells is an important underlying mechanism leading to impaired anti-tumor immunity. Emerging studies have revealed that altered amino acid metabolism is tightly linked to tumor outgrowth, metastasis, and therapeutic resistance through governing the fate of various immune cells. During these processes, the concentration of free amino acids, their membrane bound transporters, key metabolic enzymes, and sensors such as mTOR and GCN2 play critical roles in controlling immune cell differentiation and function. As such, anti-cancer immune responses could be enhanced by supplement of specific essential amino acids, or targeting the metabolic enzymes or their sensors, thereby developing novel adjuvant immune therapeutic modalities. To further dissect metabolic regulation of anti-tumor immunity, this review summarizes the regulatory mechanisms governing reprogramming of amino acid metabolism and their effects on the phenotypes and functions of tumor-infiltrating immune cells to propose novel approaches that could be exploited to rewire amino acid metabolism and enhance cancer immunotherapy.

## Introduction

Studies over the past few decades have demonstrated that malignant tumors and the immune system are intimately connected. The composition of the tumor microenvironment (TME) is complex, comprising various cell types such as immune cells, fibroblasts, and endothelial cells which exhibit intrinsic regulatory effects on tumor cells [1–4]. Immune cells, including myeloid and lymphoid cells, play critical roles in promoting inflammation and immunosurveillance on one hand; and inhibiting inflammation and enabling immune escape on the other. Some immune cells, such as helper T (Th) cells are dichotomized into Th1/Th2 subtypes. Th1 represents cells with a pro-inflammatory role, whereas Th2 cells exhibit an anti-inflammatory role. Similarly, macrophages are also dichotomized to M1/M2 subtypes. M1 subtypes promote a Th1 response and an anti-tumor function, and M2 subtypes display a Th2-like response and a pro-tumor effect. Thus, the differentiation status of immune cells may dictate their anti-tumor functions. The differentiation process of immune cells from their progenitors is directed by many factors, such as selective gene expression, cytokines stimulation, antigen presentation, nutrient metabolism and so on [5–7]. Besides governing cell fate, these factors also directly influence immune cell function. Whereas many of these factors have been extensively studied, metabolism-mediated regulatory mechanisms are still poorly understood [1, 8].

Metabolic reprogramming is a hallmark of the TME and has recently received increased attention. Various nutrients including glucose, lipids, and amino acids, play essential roles in regulating tumorigenesis through acting on both tumor cells and immune cells [9, 10]. These nutrients, as well as their metabolites, are sensed primarily by the mTOR complex through a series of biochemical networks [11, 12]. Among these major nutrients, amino acids play a dominant role in regulating mTORC1 [13]. During tumorigenesis, mTORC1 signaling regulates lipid synthesis, protein synthesis, cell survival, proliferation, and angiogenesis through targeting various substrate such as S6K1, hypoxia inducible factor 1 subunit alpha (HIF1α), and 4E-BP [11]. Collectively, mTOR functions as a central hub of nutrient signaling and cell growth across species [14]. While the roles of lipid metabolism in tumor immunity have been recently discussed [15], the regulation of amino acids metabolism in tumor immunity has not been extensively summarized. Therefore, we provide a comprehensive analysis of the roles of amino acid metabolism in regulating immune cells and their dysfunction within the TME.

The story of amino acids began from experiments carried out as early as 1827 when asparagine, isolated from asparagus juice, was hydrolyzed by Auguste-Arthur Plisson and Étienne Ossian, leading to the discovery of aspartic acid. In the subsequent 200 years, amino acids have been characterized as one of the fundamental units of life. In addition to serving as the basic building blocks of proteins, amino acid metabolism is also involved in many other cellular processes to dictate cellular functions. Given the marked increase in growth kinetics of malignant tumors, one can speculate that amino acid metabolism in the TME is significantly altered. Rewiring of amino acid metabolism in infiltrating immune cells directly impact their biological functions [16]. In this regard, amino acid transporters, sensors, and the rate-limiting metabolic enzymes are key gatekeepers of the metabolic flow. Aberrancies or deficiencies of this flow may reprogram amino acid metabolism in the infiltrating immune cells and impair anti-tumor immunity. Thus, key regulators of this pathway could be regarded as potential therapeutic targets.

Here, we briefly introduce the physiologic functions of immune cells and amino acids. We summarize the immunomodulatory effects of amino acid metabolism on various immune cell lineages under homeostatic and inflammatory settings, as well as in the TME, with an emphasis on amino acid competition between the immune cells and tumor cells. From a clinical perspective, we also discuss potential therapeutic strategies targeting amino acids metabolism pathways for improving cancer immune therapy.

## Amino acid metabolism in lymphoid cells

### T lymphocytes

T cells, or T lymphocytes, play a key role in cellular immunity and are responsible for eliminating cells expressing “foreign” antigens. With respect to cancer, once tumor cells begin to express tumor-associated antigens, specific T cells may view these cells as foreign and target them for cell death. T cells could be classified into two clusters depending on their membrane markers. The CD4^+^ cluster includes follicular helper T (Tfh) cells, T helper (Th) cells, and regulatory T cells (Treg); and the CD8^+^ cluster includes cytotoxic T cells and memory T cells. CD8 is predominantly an α/β heterodimer, but α/α homodimers are also present and recognizes endogenous antigens presented on MHCI, whereas CD4 recognizes exogenous antigens presented on MHCII. However, T cells may not be precisely defined by their cellular functions. For instance, the effector T cells (Teff) definition refers to Th cells or cytotoxic CD4^+^ and CD8^+^ T cells, which are characterized as secreting inflammatory cytokines and are cytotoxic to foreign cells [17].

**Fig. 1:**
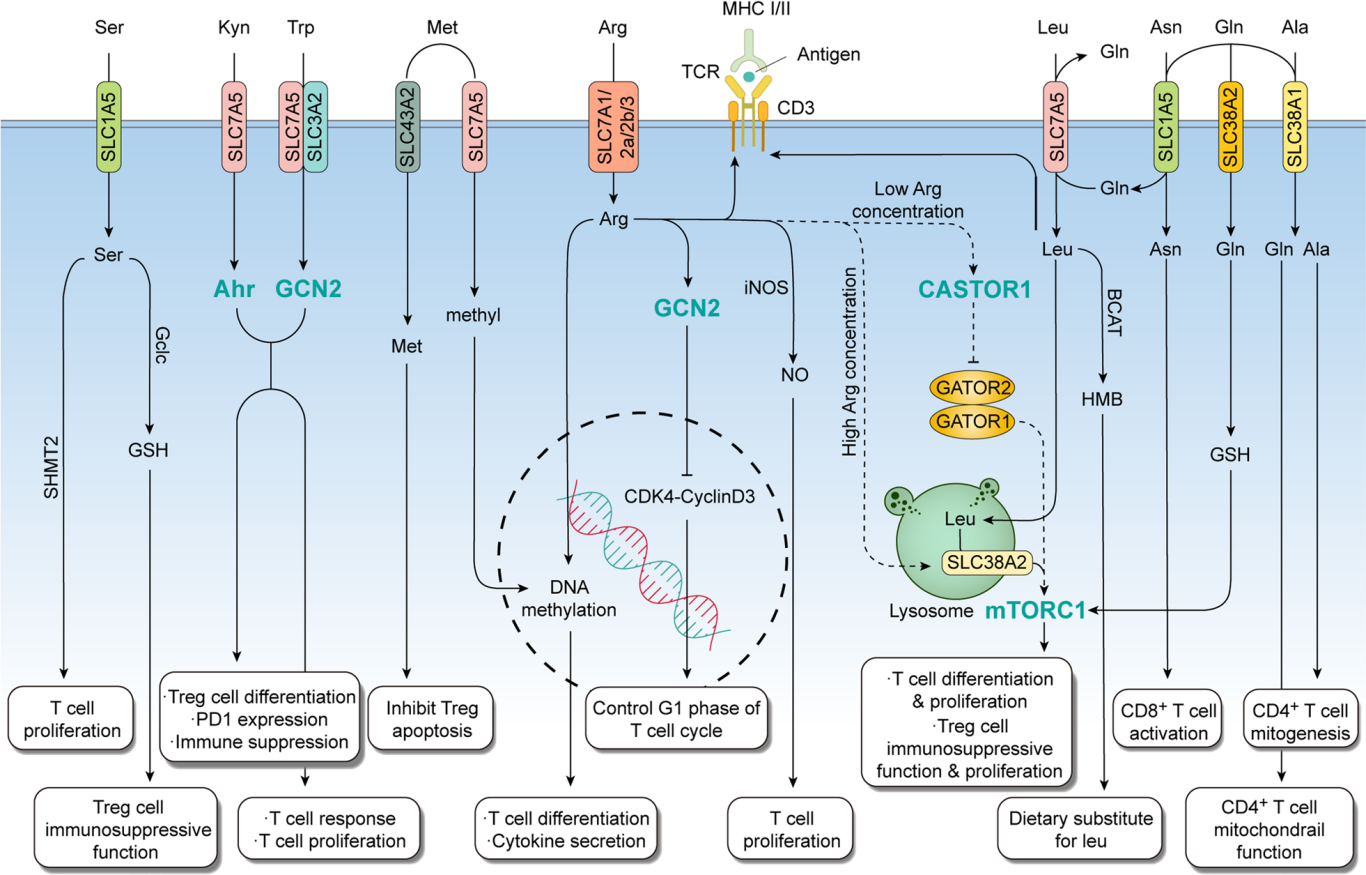
Amino acid metabolism in effector T cells. Various amino acids are transported by SLC transporters into the cytoplasm. These amino acids activate sensor proteins in the cytoplasm such as mTOR, GCN2, and Sestrin (highlighted in green bold font), directly activate the TCR-CD3 complex, or are metabolized further to affect T cell development and survival. Solid lines represent effects and reactions in T cells, and dashed lines represent uncertain effects and reactions in T cells. The arrow line represents activation and the line with a bar at the end represents inhibition. *AHR* Aryl hydrocarbon receptor; *Ala* Alanine; *Arg* Arginine; *Asn* Asparagine; *BCAT* Branched-chain aminotransferases; *Gclc* Glutamate cysteine ligase; *GCN2* General control nonderepressible 2; *Gln* Glutamine; *GSH* Glutathione; *HMB* β-hydroxy-β-methylbutyrate; *iNOS* Inducible isoform of NO synthase; *Kyn* Kynurenine; *Leu* Leucine; *Met* Methionine; *MHC* Major histocompatibility complex; *mTORC1* Mechanistic target of rapamycin kinase complex 1; *Ser* Serine; *SHMT2* Mitochondrial serine hydroxymethyltransferase; *SLC* Solute carrier; *TCR* T cell receptor; *Trp* Tryptophan

It is noteworthy that Tregs are CD4+ CD25+ T cells with immune suppressive functions that are necessary for maintaining self-tolerance [18]. Subsequent studies identified Tregs as expressing the transcriptional factor FOXP3 [19, 20]. Tregs play important roles in regulating immune surveillance and antitumor immune response through suppressing the activities of CD4+ helper and CD8+ cytotoxic T cells. Upregulation of immunosuppressive molecules including CTLA-4, PD-1, PD-L1 is essential for immune evasion during tumorigenesis. The relationships between Tregs and other T cells, as well as the roles of immune checkpoints in anti-cancer immune therapy, has been previously reviewed [21–23]. Prior studies have indicated that both CD4^+^ and CD8^+^ T cells are essential for a robust immune response and diverse amino acids play a critical role in regulating T cells (Fig. 1).

#### Branched-chain amino acids (BCAAs)

The three BCAAs are leucine, isoleucine, and valine, all of which are essential amino acids that cannot be de novo synthesized in humans. Leucine was isolated by Joseph Louis Proust in an impure form called caseous oxide in cheese in 1818 [24]. Felix Ehrlich first discovered isoleucine and isolated it from fibrin in 1904, and named it “iso-” because of its similar structure and function with leucine [25]. Emil Fischer, who was awarded the Nobel Prize in Chemistry in 1902, isolated valine from casein in 1902 [26]. In addition to serving as a basic building block of protein synthesis, under physiological conditions BCAAs are also important regulators of global cellular protein synthesis. In the normal tissue microenvironment, a study in cats found that high doses of leucine enhanced the secretion of IL-10 by T cells to inhibit the immune response [27]. Another study found reduced survival of influenza virus infected mice with high BCAA diets, which might be attributed to an increase of the number of CD8^+^ T cells accompanied with overexpression of cytotoxic cytokines resulting in tissue damage [28].

The uptake and distribution of leucine is carried out by L-type amino acid transporter (LAT), which directs amino acids across the plasma membrane in a Na+-independent manner [29]. The structure of LAT1, encoded by SLC7A5, revealed the mechanism of LAT1 amino acid selectivity [30, 31]. In T cells, antigen signaling mediated through the interaction of the T cell receptor (TCR) and LAT1 increased leucine uptake [32], which may be a self-protective mechanism for T cells to compete with cancer cells for the limited supply of amino acids within the TME. It is possible that coupling amino acid uptake with T cell signaling exists for other amino acids as well. Interestingly, LAT1 itself is a bidirectional transporter that regulate the influx of leucine and the efflux of glutamine [33], whereas another transporter, ASCT2 (encoded by SLC1A5), allows cellular uptake of glutamine [34]. However, the functions of ASCT2 and LAT1 do not appear to be coupled [35]. BCAAs are catabolized by Branched-Chain Aminotransferases (BCATs), including BCAT1 and BCAT2, also known as cytosolic branched-chain aminotransferase (BCATc) and mitochondrial branched-chain aminotransferase (BCATm), respectively. BCATs transfer amino groups from BCAA to α-ketoglutarate to produce their respective branched-chain ketoacids (BCKA) and glutamate [36, 37]. β-Hydroxy-β-methylbutyrate (HMB) is a metabolite of BCAAs, which favors a switch of Th1-type to Th2-type cells [38]. The concentration of extracellular L-arginine controls the expression of the T cell antigen receptor ζ chain (CD3ζ), to influence the antigen recognition function of T cells [39, 40].

Besides its effects on the TCR, another well characterized function of leucine is regulating the mTOR pathway. mTOR is found in two complexes, mTORC1 and mTORC2, which have different sensitivities to rapamycin and are composed of distinct subunits. Several reviews have covered the functions of mTOR on downstream T cell activation, differentiation, and homeostasis [41–43]. Expression of BCATc is induced by TCR signaling in CD4+ T cells. BCATc^−/−^ mice have increased levels of leucine which enhances mTOR activity, leading to increased phosphorylation of downstream substrates including S6 and 4EBP-1 [44]. Sestrin2, SAR1B, and GATOR2 are all the upstream regulators of mTOR. Both Sestrin2 and SAR1B are bonded to GATOR2 to inhibit mTORC1 signaling, and their interactions with GATOR2 can be disrupted by leucine, indicating a role for Sestrin2 and SAR1B as a sensor of leucine to activate mTOR activity [45, 46]. However, the function of these two sensors has some differences. For instance, Sestrin2 may show increased sensitivity to high concentration of leucine, whereas SAR1B may sense lower levels of leucine to maintain basal mTOR activity [46]. Although Sestrin2 and SAR1B have been studied in other cell types, they likely carry out important functions in T cells as well. While leucine controls mTOR activity to regulate T cell proliferation and differentiation, it does not appear to be required for the maintenance of naïve T cells [32].

However, mTOR activity is also essential for the immune suppressive Tregs. Treg-specific mTOR knockout mice revealed mTOR was critical for Treg differentiation, activation, and migration into non-lymphoid tissues [47]. A role for amino acids in regulating Treg function and immune tolerance through mTOR was further demonstrated to be dependent on RagA/B and Rheb1/2 GTPases where arginine and leucine functioned to induce and sustain mTORC1 activity in Treg cells [48]. Results such as these suggest that factors such as RagA/B and Rheb1/2 may serve as potential therapeutic targets. In addition to mTORC1, the cooperation of mTORC1 and mTORC2 also contributes to the maintenance of Tregs suppressive activity, but mTORC2 was not necessary for Tregs function [49]. Similarly, systemic lupus erythematosus, an autoimmune disease, exhibited increased activity of mTORC1 and mTORC2, but diminished Treg suppressive activity [50]. Therefore, the role of amino acids in activating mTOR and its role in controlling Tregs needs further exploration.

BCAA metabolism is hyperactive in multiple malignancies including leukemia, lung cancer, bone sarcomas, hepatic carcinoma, pancreatic ductal adenocarcinoma, and colorectal cancer [51–57]. Although there is no direct evidence demonstrating a role of BCAA metabolism in anti-tumor immunity, these observations indicate that activation of BCAA metabolism might be linked to dysfunction of tumor-infiltrating T cells.

#### Glutamine

Glutamine was first discovered by Schulze in 1883, with the synthesis of glutamine further described by Hans Krebs in 1935, and its importance in organismal homeostasis was explored in animal experiments [58]. Subsequently, research on glutamine in the fate of cancer cells and cancer therapy has been extensive, but its role in tumor-associated immune cell function is just emerging [59].

Overexpression of SLC38A1, a glutamine transporter, markedly enhanced mitochondrial function in human CD4^+^ T cells exposed to ascites derived from patients with ovarian cancer, a process shown to be associated with glucose metabolism [60]. Another glutamine transporter, SLC38A2, was found to be critical for the generation and memory T cells in part through modulating mTORC1 activity [61]. Furthermore, the proliferation and function of Tregs is dependent on the expression of SLC7A11, which is controlled by the nuclear factor erythroid 2-related factor 2 (NRF2) [62]. However, this study did not explore the exact substrate effects on Tregs regulation. Although SLC7A11 is regarded as an antiporter of cysteine and glutamate transporter, this study did not explore the exact substrate effects on Tregs regulation. Moreover, SLC7A11 is highly expressed in tumor cells where it imports cystine for GSH biosynthesis and antioxidant defense. This effect is partly due to suppression of ferroptosis, another powerful tumor suppression mechanism that may eliminate precancerous cells exposed to metabolic stress and nutrients starvation [63]. Thus, these observations support the notion that SLC7A11 may serve as an additional mechanism specific in tumor or immunosuppressive cells to capture free cystine in the TME to promote tumor growth and attenuate the function of anti-tumor immune cells. However, the role of SLC7A11 in anti-tumor immune cells needs further investigated.

As the precursor of protein O-GlcNAcylation, glutamine is involved in regulating T cell self-renewal [64]. Glutamine maintains Teff cell ATP concentrations through glutamine-dependent mitochondrial metabolism [65]. The catabolism of glutamine promotes de novo synthesis of glutathione (GSH) to impact T cell differentiation [66], a process that also involves mTOR activity [67, 68].

Despite glutamine regulating T cell activation and function, blocking glutamine metabolism does not weaken T cell function as anticipated, but rather enhances T cell antitumor activity [69]. One possible reason behind this observation is that tumor cells are addicted to glutamine, and thus glutamine starvation may have more direct consequences on tumor cell survival than T cells.

#### Tryptophan

L-tryptophan was discovered in 1901 by Frederick Hopkins and Syndey Cole from the lysate of casein digested by insulin [70]. And it was subsequently identified as one of the essential amino acids in 1912 by Hopkins [71]. Although a small fraction of free tryptophan is used in protein synthesis and to produce neurotransmitters such as serotonin, over 95% of free tryptophan is degraded through the kynurenine pathway [72], which provides metabolites such as melatonin, quinolinic acid, kynurenine, tryptamine, vitamin B3, nicotinamide adenine dinucleotide (NAD+), and nicotinamide adenine dinucleotide phosphate (NADP+).

Under physiological conditions, enzymes in the tryptophan-kynurenine pathway are constitutively expressed, thus tryptophan metabolism has widespread effects, from central excitatory neurotransmission to peripheral immune response and inflammation [73]. The function of tryptophan in T cells mainly serves as an immune inhibitory factor. In general, tryptophan is catabolized to kynurenine by Indoleamine 2,3-dioxygenase (IDO), which is then further catabolized by other kynureninases [72]. Besides IDO, another tryptophan-degrading enzyme, tryptophan-2,3-dioxygenase (TDO2), is expressed in the liver and catalyzes tryptophan to kynurenine [74]. While tryptophan conversion to kynurenine has not been shown to take place in T cells, this pathway functions in other cells within the TME including macrophages and tumor cells, thus tryptophan metabolism may indirectly influence T cell function.

In normal tissue environments, IDO activity has been directly associated with suppressing the immune response. During pregnancy, IDO prevented the fetal alloantigen response by inhibiting T cell-driven local inflammation [75], possibly due to attenuation of central and effector memory CD8^+^ T cell generation, an effect that was lost once these T cells were present [76]. During human hematopoietic stem-cell transplantation, the upregulation of extracellular kynurenine effectively suppressed T cell responses and promoted apoptosis of Teff cells [77]. Moreover, the ratio of tryptophan and kynurenine may be used as a predictive marker of CD4^+^ T cell populations [78]. Besides kynurenine, other metabolites of tryptophan also function in suppressing immune function. In a rat cardiac allograft model, 3-hydroxyanthranilic acid markedly prolonged survival [79], which may serve as a model of allograft transplantation induced immune tolerance. Interestingly, both kynurenine and picolinic acid inhibited T cell proliferation, but had no effect on the activation of resting T cells [80]. Furthermore, while 3-hydroxykynurenine and 3-hydroxyanthranilic acid inhibited T cell responses, anthranilic and quinolinic acid had no effect [76].

System L, a heterodimer composed of a heavy chain of CD98 (encoded by SLC3A2) and one of two catalytic L chains LAT1 (encoded by SLC7A5) or LAT2 (encoded by SLC7A8), transports tryptophan [81]. With low tryptophan levels in the TME, tumor cells may use two mechanisms to enhance tryptophan uptake to outcompete Teff cells for tryptophan. One mechanism may be for tumor cells to secrete toxic metabolites such as kynurenine to attenuate neighboring Teff cells anti-tumor function [82]. Another mechanism could be that system L is not a unique transporter for tryptophan, and another specific tryptophan transporter is induced in IDO expressed tumors [83]. Moreover, general control nonderepressible 2 (GCN2) kinase could serve as an intracellular amino acid sensor capable of phosphorylating eukaryotic translation initiation factor 2-alpha (eIF2α) to lower global protein synthesis rates in response to amino acid starvation [84]. Tryptophan depletion leads to T cell anergy and proliferation arrest, and disruption of GCN2 might prevent T cells from IDO-mediated inhibition [85].

Prior studies have shown that activation of GCN2 could inhibit inflammatory Th17 and Th9 cell differentiation [86, 87], potentially through GCN2-mediated activation and downregulated of key enzymes involved in fatty acid synthesis, which is a prerequisite of CD4^+^ T cell proliferation and differentiation, suggesting that GCN2 activity may weaken the immune response [88]. However, other studies have observed that GCN2 is essential for T cell function. For instance, GCN2 deficient T cells fail to proliferate when amino acids are limiting [89], and knockout of GCN2 impaired many aspects of T cell immunity in brain tumors [90]. In addition, both IDO and TDO inhibitors prevent the accumulation of immunosuppressive tryptophan catabolites but do not enhance T cell responses via rescuing GCN2 from tryptophan starvation, thus GCN2 might not play an important role in the response to tryptophan starvation [91]. Therefore, these varied conclusions reveal that the relationship between tryptophan metabolism and T lymphocytes remain unclear and worthy of further investigation.

Tryptophan metabolism also leads to the production of GCN2-dependent Tregs [92]. Kynurenine activated aryl hydrocarbon receptor (AhR)-expressing T cells to differentiate into Tregs but not AhR-null T cells, and TGF-β upregulated AhR expression to potentially induce the generation of Tregs [93]. Kynurenine is transported across the plasma membrane of T cells by SLC7A5 or SLC7A8 and sensed by AhR in the cytoplasm [82, 94] to upregulate PD-1 expression in CD8^+^ T cell, driving the generation and differentiation of Tregs [95, 96]. Inhibition of AhR was shown to suppress IDO/TDO mediated tumor progression, which synergizes with PD-1 blockade [95]. These studies indicate that the immunosuppressive axis, try-kyn-AhR, may participate in tumor progression through regulating Tregs.

#### Methionine

Methionine, initially named sulfurous amino acid, was isolated by J. H. Muller in 1923 [97]. Unfortunately, he made an incorrect summation formula. Three years later, his colleague S. Odake corrected that formula and named this amino acid methionine [98]. In 1928, the structure of methionine was solved by G. Barger and F. P. Coyne [99]. Methionine is another essential amino acid, and as with other amino acids, its primary function is in protein synthesis to satisfy T cell proliferation. Methionine also serves as a critical source of methyl groups in cells through the methionine cycle, during which methionine is converted into S-adenosylmethionine (SAM) by methionine adenosyltransferase (MTA), to participate in many important biochemical processes, including float metabolism, redox maintenance, polyamine synthesis, and as the methyl donor molecule for all methylation reactions [100]. In addition, methionine is also crucial for the production of endogenous hydrogen sulfide [101] and plays a role in inhibiting autophagy [102]. A recent study discovered that methionine also provides methyl groups for DNA and RNA methylation, which further promote differentiation and proliferation of T cells [103].

The importance of methionine in T cells is not dependent on differences between intracellular and extracellular methionine concentration gradients, but rather T cells upregulate the expression of the methionine transporter SLC7A5 following stimulation of with antigen. Based on this, SLC7A5 is recognized as the rate-limiting factor for the generation of methyl groups during T cell activation [103]. However, methionine import varies in tumor cells. Besides SLC7A5, the transporter SLC43A2 is also involved in methionine uptake in tumor cells allow them to outcompete T cells for limited methionine availability [104]. Following import of methionine, intracellular methionine regulates cellular functions within the nucleus. Methionine restriction reduces histone H3K4 methylation (H3K4me3) on promoters of key genes involved in Th17 cell proliferation and cytokine production, and exposing mice to a diet deficient in methionine reduced the expansion of pathogenic Th17 cells, leading to a reduction of T cell-mediated neuroinflammation and disease [105, 106]. In addition, tumor cells outcompete T cells for methionine leading to a decrease of intracellular methionine and SAM levels in CD8+ T cells reducing H3K79me2 and STAT5 gene expression to attenuate immune function [104]. There is also direct evidence revealing a relationship between methionine levels and anti-tumor function of T cells. In this context, elevation of SAM and its downstream catabolites 5-methylthioadenosine reprogrammed the global chromatin accessibility of CD8^+^ T cells, which attenuated T cell anti-tumor function [107]. Moreover, the role of SLC43A2 was also shown to be critical for the survival of Tregs, as the downregulation of SLC43A2 increased the Treg apoptosis due to the decrease of methionine uptake [108].

#### Arginine

Since the discovery of arginine from lupin seedling extracts in 1886 by Schulze and Steiger [109], studies of arginine focusing on its basic functions and clinical relevance have been of high interests over the past century. Arginine is a nonessential amino acid, but the de novo synthesis of arginine does not satisfy the daily demand of the human body. Thus, arginine is defined as a semi-essential, or conditionally essential. Under physiological conditions. Arginine is mainly metabolized by arginase-1 (Arg1) and nitric oxide synthase (NOS) in myeloid cells, generating urea and L-ornithine, and NO and L-citrulline, respectively [110].

Studies have found that reduction of arginine attenuates T cell function and proliferation, which can be reversed by arginine supplementation [111–114]. Using a mouse model of sepsis, arginine supplementation contributed to the maintenance of CD4^+^ T cells in the blood and para-aortic lymph node, but this was abrogated by inhibition of NOS, indicating that production of NO through NOS was important for proliferation of T cells [115]. Under arginine restriction, T cells downregulate CD3ζ expression, preventing the expression of the TCR leading to reduced proliferation and cytokine secretion [116]. In neonates, arginine might regulate DNA methylation to modulate the maturation of the immune system [117]. The arginine transporter, cationic amino acid transporter-1 (CAT1), supports both naïve and memory CD4^+^ T cells as well as CD8^+^ T cells to maintain T cell proliferation and activation [118].

More specifically, the uptake of arginine is controlled by the cationic amino acid transport (CAT1, 2a, 2b and 3, encoded by SLC7A) [119, 120], which function as monomers on the plasma membrane [120]. Generation and persistence of memory T cells, but not effector T cells, can be reprogrammed by arginine transported by SLC7A1 in a mechanism partially dependent on mTORC1 [61]. SLC38A9, a lysosomal transmembrane protein, serves as an intracellular arginine sensor to interact with lysosome membrane localized Rag GTPase and Ragulator, which acts as a scaffold to tether Rag GTPase and mTORC1 to the lysosome [121, 122]. Another key factor, CASTOR1, also senses arginine. However, the mechanism by which CASTOR1 and SLC38A9 sense arginine appears to be different. Arginine stimulates SLC38A9 to efflux essential amino acids, including leucine, from lysosomes to the cytosol to be recycled, but SLC38A9 has low affinity for arginine transport and minor effects on arginine concentration [123]. On the contrary, CASTOR1 binds to cytoplasmic arginine with high affinity [124]. Thus, SLC38A9 transmits an arginine sufficiency signal to mTORC1, leading to mTORC1 activation, and the overexpression of SLC38A9 partially maintains mTORC1 activation upon amino acid deprivation [122, 125]. Whereas CASTOR1 serves to transmit an arginine deprivation signal. When arginine is deprived, CASTOR1 and GATOR2 bind together promoting the dissociation of the GATOR1/GATOR2 complex (an upstream regulator of mTORC1), leading to the inhibition of mTORC1 activity [124, 126]. Moreover, the function of CASTOR1 is similar to Sestrin2 and SAR1B described above, except that they sense different amino acids. Therefore, SLC38A9 and CASTOR1 play a counterbalancing role in arginine sensing to prevent abnormal activation of mTORC1, which may play a role in regulating proliferation and differentiation of tumor-associated immune cells. Supporting this notion, L-arginine depletion dampened proliferation of T cells through GCN2, which may arrest T cells in the G1 phase of the cell cycle through impairment of cyclin D3 and cyclin-dependent kinase 4 (cdk4) activity [127, 128]. Interestingly, other studies have revealed that arginine levels might influence the cell cycle through regulating the mTOR pathway. In this context, arginine deprivation suppresses the activity of mTORC1, whereas the activity of mTORC2 increases, leading to cell cycle arrest, which can be reversed by arginine supplementation [129]. As important amino acid sensors, both GCN2 and mTOR signaling pathways regulate cell cycle progression of T cells. Regarding therapeutic T cells, such as chimeric antigen receptor (CAR)-T cells, recent evidence suggests that low arginine levels also impair their proliferation [130].

As a semi-essential amino acid, the de novo synthesis of arginine also plays an important role in maintaining intracellular arginine concentrations. Argininosuccinate synthetase (ASS) and argininosuccinate lyase (ASL) are key enzyme in arginine synthesis. ASS catalyzes condensation of citrulline and aspartate to argininosuccinate which is then converted to arginine and fumarate by ASL [131]. Citrulline, which is transported by SLC7A5 (LAT1), could partially substitute for arginine deficiency to promote T cell proliferation via ASS and ASL in T cells, whereas rescue of proliferation was not observed in complete absence of arginine [132].

Besides reprogramming of amino acid metabolism, additional studies have found that other metabolic pathways such as glucose metabolism also regulates the activity of Tregs through the mTOR pathway [49, 133]. Tregs are characterized by oxidative and catabolic metabolism. However, Toll-like receptor (TLR) signals promote Treg proliferation and increased glycolysis, anabolic metabolism, and expression of glucose transporter Glut1. Glut1 reduced expression of *FOXP3* in Tregs and hampered their immunosuppressive capacity [134]. Thus, mTOR plays a key role in regulating amino acid, lipid, and glucose metabolism in Tregs, with further studies likely to uncover regulation of other metabolic pathways by mTOR in Tregs.

In the TME, owing to a lack of Arg1 and NOS in T lymphocytes, arginine may be consumed by Arg1 and NOS in tumor cells or myeloid cells leading to excessive arginine consumption and a weakening of the Teff immune response. Besides the direct regulation on T cells, L-arginine depletion induced myeloid-derived suppressor cells (MDSCs) to blunt the anti-tumor response of Teff cells [135]. Therefore, arginine depletion caused by elevated Arg1 in the TME has been considered a hallmark immunosuppressive mechanism. However, recent studies revealed Arg2, an isoform of Arg1 which functions in the mitochondria, is expressed in T cells and serves as a regulator of CD8^+^ T cell activation, anti-tumor cytotoxicity, and memory formation, independently of extracellular arginine concentration [136]. L-arginine is rapidly converted into downstream metabolites, and the addition of the arginase inhibitor norNOHA reduces this conversion, indicating that arginine is catabolized by Arg2 in T cells [137]. When Arg2^−/−^ CD8^+^ T cells and wild-type CD8^+^ T cells were co-transferred into wild-type mice, Arg2^−/−^ CD8^+^ T cells outcompeted wild-type cells suggesting that Arg2 was detrimental to T cell survival [137]. This may partially explain why Arg2^−/−^ CD8+ T cells suppress tumor growth and synergize with PD-1 blockade [136]. Therefore, Arg1 or Arg2 are both negative regulators of T cell survival and function with Arg1 existing in cells other than T cells to consume free arginine in the TME, and Arg2 is found in T cells to consume intracellular arginine. Both intracellular and extracellular deprivation of arginine are essential means to regulate the proliferation of T lymphocytes and maintain tissue homeostasis.

#### Serine

In 1865, Emil Cramer extracted component from silk, and named it serine from the Latin of silk, *sericum* [138]. As a non-essential amino acid, serine is synthesized de novo from the glycolytic intermediate 3-phosphoglycerate or transported by several transporters such as SLC1A5 into cells that cannot synthesize serine like mature neurons [139, 140]. Serine supports metabolic processes including protein and glutathione synthesis and serves as a crucial one-carbon donor for the folate cycle to participate in nucleotide synthesis, methylation reactions, and generation of NADPH [141, 142]. Based on these roles of serine, targeting cancer-associated serine metabolism is viewed as a potential cancer therapy.

Generally, following antigenic stimulation, the rate of serine synthesis increases in activated T cells to provide intracellular glycine and one-carbon metabolites to support the proliferation of T cells, indicating that serine is essential to the T cell adaptive immune response, and extracellular serine is dispensable to support optimal T cell expansion even when glucose is sufficient for T cell activation and function [143]. Another study found that in order to adapt a state of rapid proliferation of T cells, naïve T cells induce the reprograming of mitochondrial biogenesis and modeling, with one-carbon metabolism being the most highly induced pathway, which is fed by serine, and disruption of the key mitochondrial serine hydroxymethyltransferase (SHMT2) inhibiting T cells proliferation [144].

In the TME, serine activates mTOR to inhibit *FOXP3* expression and further hinder Tregs immunosuppressive capacity, but the transformation of serine to glutathione through glutamate cysteine ligase (Gclc) contributed to preserve Tregs immunosuppressive function, and Gclc ablation showed autoimmunity and enhanced anti-tumor responses [145].

Given serine is a non-essential amino acid, relatively fewer studies have focused on its role in immunity. However, with the studies described above highlighting a key role for serine in T cell function, a role for serine metabolism in the tumor-associated immune response should be assessed further.

### B lymphocytes

**Fig. 2:**
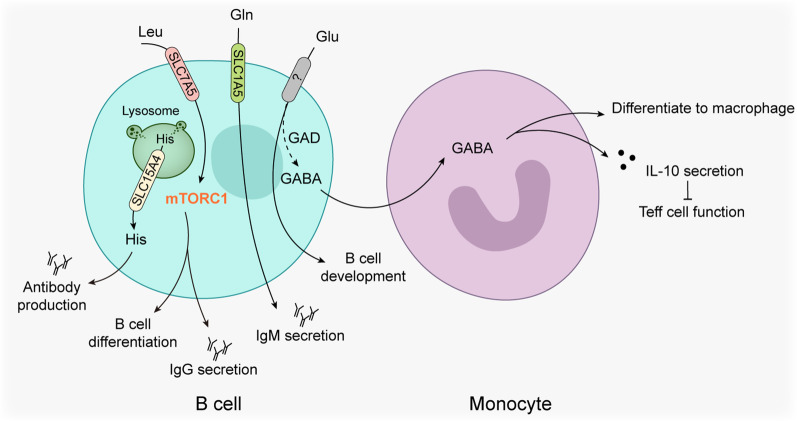
Amino acid metabolism in B cells. Several amino acids are associated with B cell function and differentiation, and threonine can further affect the differentiation and function of monocytes. The solid line represents certain effects and reactions in B cells, and the dashed line represents potential effects and reactions in B cells. The arrow line represents activation and the line with a bar at the end represents inhibition. *GABA* γ-aminobutyric acid; *GAD* Glutamate decarboxylase; *Glu* Glutamate; *His* Histidine; *IgG* Immunoglobulin G; *IgM* Immunoglobulin M; *IL* Interleukin; *Leu* Leucine; *mTORC1* Mechanistic target of rapamycin kinase complex 1; *SLC* Solute carrier; *Teff cell* Effector T cell

B cells have been recognized as a core component of humoral immunity. The mechanism by which B cells serve to defend against foreign antigens occurs through three different pathways: antibody secretion, antigen presentation, and their direct killing capacity. Following stimulation, B cells differentiate into plasma cells, and the immunoglobulins secreted by plasma cells facilitate the immune responses by antibody-dependent cellular cytotoxicity (ADCC) and complement-dependent cytotoxicity (CDC). Several studies have revealed that a subset of amino acids play a role in these important processes (Fig. 2).

Leucine, transported into B cells through SLC7A5, targets mTORC1 to promote B cell differentiation, as well as supports the production of IgG and cytokines [146]. Furthermore, inhibition of the glutamine transporter SLC1A5 or key enzymes of glutamine metabolism, led to reduced production of IgM in B cells [147]. Moreover, restriction of amino acids such as tryptophan led to a developmental arrest of B cells in the bone marrow [148]. In addition, histidine is transported by SLC15A4 from the lysosome to cytosol, and loss of SLC15A4 disturbed the Toll-like receptor 7-triggered, mTOR-dependent IRF7-IFN-I circuit that leads to auto-antibody production [149]. Given this importance of amino acids in B cell function, further studies are necessary to fully understand a role for amino acids in regulating B cell function.γ-Aminobutyric acid (GABA) originates from glutamic acid, one of the non-essential amino acids, through key enzymes including glutamate decarboxylase (GAD). In the past, GABA has been extensively studied in the context of neurobiology as a central suppressive neurotransmitter. Interestingly, GABA has also been shown to play an important role in B cells anti-tumor immunity. GABA is synthesized and secreted by activated B cells and plasma cells, and this GABA further promotes monocytes to differentiate into anti-inflammatory macrophages that secrete interleukin-10 and inhibit CD8^+^ T cell tumor killing function [150]. In addition, tumor cells aberrantly expressing GAD1 could enhance the tumors immune suppressive function through autocrine action of GABA [151]. Thus, GABA secreted by B cells may directly target tumor cells to promote tumor growth, therefore GAD should be studied as a potential anti-cancer target.

Germinal centers (GCs) are components of secondary lymphoid organs (SLOs) within the spleen, lymph nodes and mucosa-associated lymphoid tissue (MALT). They have important sites of B cell clonal expansion, somatic hypermutation, and affinity-based selection, a process that leads to the production of high-affinity antibodies [152]. GCs are divided into a light zone (LZ) and dark zone (DZ) which harbor distinct functions. The DZ is the site of B cell clonal expansion and somatic hypermutation, whereas the LZ is the region where B cells undergo positively selection for affinity-based mutations with the help of T follicular helper (Tfh) cells [153]. Rapamycin, an mTOR inhibitor, has been found block the formation of GCs [154]. Deficiency of the mTORC1 subunit Raptor reduce the population of B cells in GCs [155], and Raptor has been shown to fuel B cells differentiation and proliferation early after positive selection in the LZ, whereas it only regulates proliferation in the DZ [156, 157]. B cell homeostasis and function also requires Rictor, a subunit of mTORC2 [158]. Both mTORC1 and mTORC2 guard Tfh phenotypic and functional maturation to support B lymphocytes development [159, 160]. In addition, glucose, lipid and hypoxia influences GCs B cells through the metabolic sense of mTOR [161–163], and some chemokines such as BCL-6 [164] and CCL2 [165] are important regulators of the mTOR pathway in GC B cells. In Peyer’s patches (PPs), another SLO, nutrient deficiency drastically reduces secretion of CXCL13 from stromal cells via PI3K-Akt-mTOR axis to decrease the number of B cells in SLOs, and further attenuate the digestive immune system [166]. In addition, the PI3K-Akt-mTOR axis is also be activated by CXCL13/CCR5 to promote proliferation and migration of clear cell renal cell carcinoma [167]. Although this study did not explore the functional consequences to B cells or nutrient deprivation, one may speculate that nutrients could affect tumor progress through the regulation of B cells. Because B cells are critical for tertiary lymphoid structures (TLS), a tumor infiltrated ectopic lymphoid organ similar to SLOs, multiple studies found the higher number of infiltrating B cells in TLS indicate a better patient survival with malignant tumors, however the mechanism remains unclear [168, 169].

In summary, although several amino acids such as leucine, glutamine and glutamic acid have been shown to regulate tumor-associated B cells, the importance of mTOR in the formation of TLS and a correlation with the presence of B cells implicated within TLS suggests a key role of amino acids in both general biology and anti-tumor roles of B cells that deserves further investigation.

### Natural killer cells

**Fig. 3:**
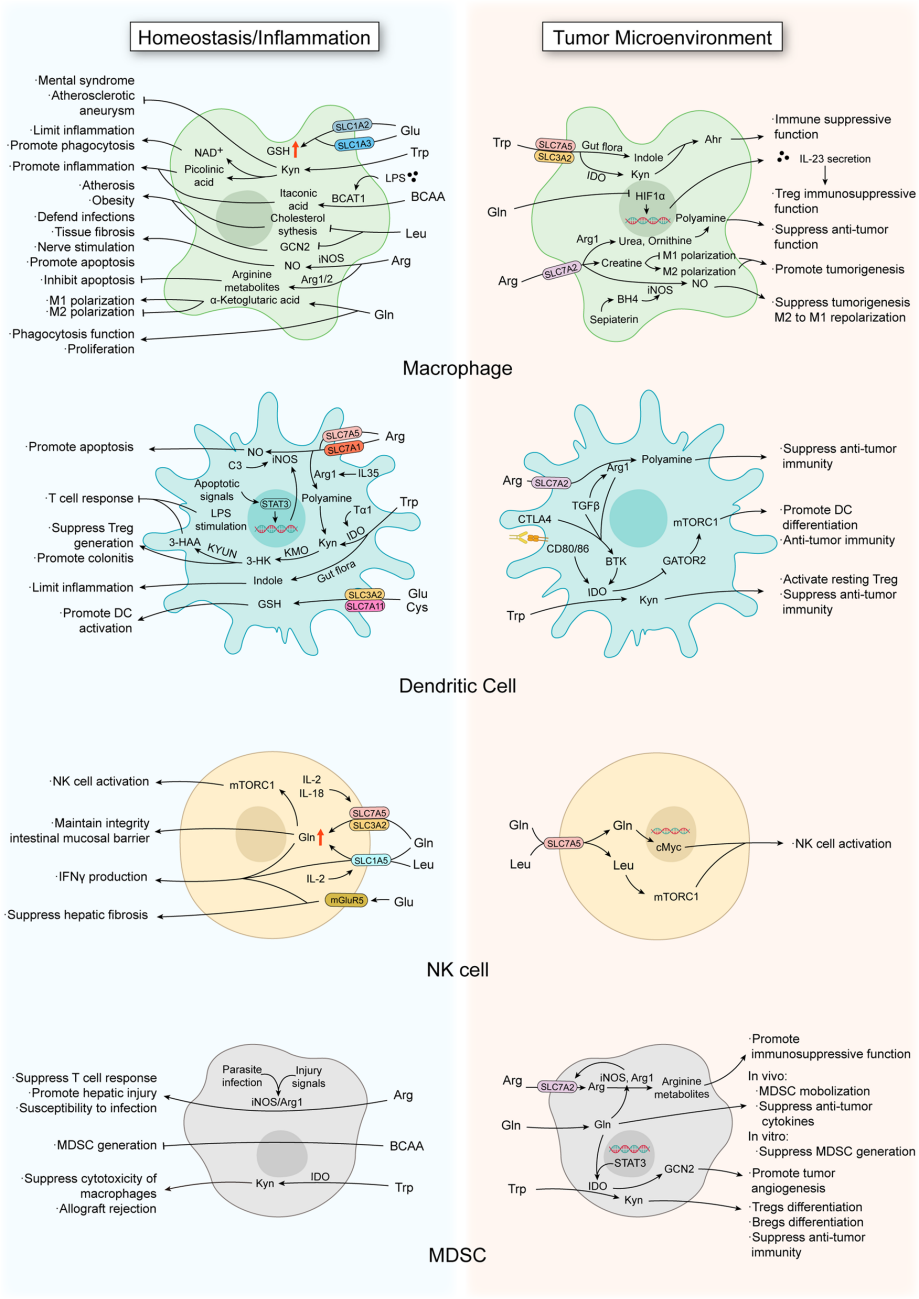
Reprogramming of amino acid metabolism in the innate immune system. In the innate immune system, various amino acids affect innate immune cell functions. The same amino acids may play different role in the homeostasis/inflammation microenvironment and tumor microenvironment. The arrow line represents activation and the line with a bar at the end represents inhibition. *3-HAA* 3-hydroxyanthranilic acid; *AHR* Aryl hydrocarbon receptor; *Arg* Arginine; *Arg1/2* Arginase-1/2; *BCAA* Branched-chain amino acids; *BCAT1* branched-chain aminotransferases 1; *BH4* Tetrahydrobiopterin; *BTK* Bruton’s tyrosine kinase; *C3* Complement 3; *CTLA4* Cytotoxic T-lymphocyte associated protein 4; *Cys* Cystine; *GCN2* General control nonderepressible 2; *GCN2* General control nonderepressible 2; *Gln* Glutamine; *Glu* Glutamate; *GSH* Glutathione; *HIF* Hypoxia inducible factor; *IDO* Indoleamine 2,3-dioxygenase; *IL* Interleukin; *iNOS* Inducible isoform of NO synthase; *KMO* Kynurenine 3-monooxygenase; *Kyn* Kynurenine; *KYNU* 2-amino-4-[3-hydroxyphenyl]-4-hydroxybutanoic acid; *Leu* Leucine; *LPS* Lipopolysaccharide; *SLC* Solute carrier; *STAT3* Signal transducer and activator of transcription 3; *TAM* Tumor-associated macrophages; *TGF* Transforming growth factor; *Trp* Tryptophan; *Tα1* Thymosin α1

In 1975, Rolf Keissling and colleagues identified a new immune cell type mediating direct cytotoxicity against alloantigen and tumor-associated antigens, different from T or B lymphocytes [170, 171]. Subsequent studies of this cell type led to its classification as an innate lymphoid cell (ILC) named natural killer (NK) cells. Using high-throughput single-cell RNA-seq, NK cells have been further broken down into two major subsets based on marker expression. The first being NK1, which are CD56^dim^ in humans and CD27^−^CD11b^+^ NK cells in mice. NK2 cells are CD56^bright^ in humans and CD27^+^CD11b^−^ NK cells in mice [172]. NK cells are widely distributed throughout the human body, including lymphoid tissues such as bone marrow, lymph nodes, and spleen, as well as peripheral tissues such as liver, skin, intestine, and uterine [173]. Thus, it is important to explore the regulating factors, including amino acids, involved in controlling NK cell functions (Fig. 3).

Glutamine has been shown to be important in supporting physiological functions of NK cells. As an important regulator of the intestinal mucosa, glutamine supplementation increases the number of infiltrating NK cells and improves barrier function in a rat model of colitis [174]. In humans, glutamine supplementation in athletes with heavy load training led to an increase in NK cells activity [175]. However, another study provided contrary results regarding glutamine in regulating NK cell activity and function, where glutamine supplementation prevented the decline in plasm glutamine concentration but had no effect on NK cell cytotoxic activity [176]. However, in tubulointerstitial fibrosis, activated transglutaminase 2 in NK cells played an exacerbating role [177].

NK cells execute anti-tumor functions in multiple ways. First, they can recognize and respond to cancer cells lacking MHCI expression [178]. ADCC is another important mechanism by which NK cells function to restrict tumor growth and survival, and thus several studies have sought out ways to enhance ADCC such as assessing synergistic effects with anti-PD-L1, cytokine stimulation, and intercellular interactions [179–181]. NK cells also produce pro-inflammatory cytokines such as IFNγ and TNFα to promote anti-tumor activity of other immune cells or to directly inhibit tumor cells. Given NK cell characteristic ex vivo activation, expansion, genetic modification, and tumor cell recognition, mechanisms to improve their tumor-killing capacity for therapeutic purposes have been studied [182], and the metabolism of amino acids has been shown to play a role.

Recent studies have shown that SLC7A5 was the predominant system L-amino acid transporter in activated NK cells, and glutamine transported by SLC7A5 increases the expression of the transcription factor c-Myc through mTORC1 activation [183]. IL-12 induces the expression of the high-affinity IL-2 receptor CD25, and several amino acid transporters including SLC7A5, SLC1A5, and SLC3A2 are upregulated in response to IL-2 stimulation [183, 184]. NKG2D-mediated IFN-γ production and degranulation were decreased when SLC1A5 and SLC3A2 where inhibited [184], but whether this is through reducing amino acid uptake remains unclear. Besides IL-2 and IL-12, other cytokines such as IL-18 were shown to facilitate SLC7A5/SLC3A2 expression to promote proliferation of NK cells through the mTORC1 pathway [185]. mTORC1 and mTORC2 are both essential for NK cell activity, but through different mechanisms. mTORC1 appears to function in early NK cell development, whereas mTORC2 may function in terminal maturation, and thus they may cooperate in a non-redundant manner [186, 187]. Specifically, mTORC1 sustains mTORC2 activity through CD122-mediated IL-15 stimulation; however, mTORC2 negatively regulates mTORC1 function through the inhibition of STAT5-mediated SLC7A5 expression [186]. Another membrane protein, metabotropic glutamate receptor 5 (mGluR5), was found to enhance cytotoxicity of NK cells following activation by glutamate, and pharmacological activation of mGluR5 accelerated the regression of liver fibrosis [188].

While studies to date have focused largely on glutamine, amino acid transporters, and mTOR signaling in NK cells, it is likely that other amino acids play a role in this axis as well, such as arginine and leucine as key mTOR regulators, which needs further investigation.

## Amino acid metabolism in myeloid cells

### Macrophages

Macrophages are important innate myeloid cells and a major cell type within the mononuclear phagocyte system (MPS) [189]. Tissue-resident macrophages have various names tied to their specific function including microglia, Kupffer cells, alveolar macrophages, and osteoclasts [190-193]. In physiological conditions, tissue-resident macrophages play an important role in tissue homeostasis and organ development. The role of macrophages in development is often overlooked, including branching morphogenesis, neuronal patterning, angiogenesis, bone morphogenesis, generation of adipose tissue, and testicular organogenesis [194, 195]. As for homeostasis, Kupffer cells efficiently phagocytize pathogens entering from the portal or arterial circulation [191], and are essential for cholesterol transport to link lipid metabolism in the liver [196]. Alveolar macrophages play an important role in inhibiting pro-inflammatory responses to tissue debris or to innocuous antigens [192]. Osteoclasts are the only cells known to resorb bone to refresh or repair the skeletal system [193]. Spleen macrophages localize to the marginal zone and red pulp, which promote T cell immune responses and recycle iron elements from defective blood cells, respectively [197]. Testicular macrophages are known to contribute to spermatogenesis and the production of male hormones outside of their classical immune function [195].

Macrophages are important innate immune cells for tissue homeostasis and organ development under physiological conditions. It is generally recognized that M1/M2 polarized macrophages are two extremes within the immune response and are induced by various internal or external signals. These polarized macrophages further coordinate with diverse factors to regulate the outcome of pathogen infection, local tumor immune responses, and tissue repair and remodeling. In fact, M1-like and M2-like tumor-associate macrophages (TAMs) exist in the same tumor, but during different stages. They play an anti-tumor role through recognizing and eliminating tumor cells; or a pro-tumor role by promoting tumor growth, invasion, and metastasis. Such phenomenon is a so-called double-edged sword, and the function or polarization of macrophages is affected by many factors including amino acids and amino acid metabolites (Fig. 3) [198].

#### Tryptophan

Tryptophan regulates macrophages in many diseases besides cancer. Low levels of free tryptophan in serum may be associated with decreased IDO activity in macrophage and mental health syndromes such as anxiety and depression in patients with chronic hepatitis C [199]. Tryptophan-containing hexapeptide-coated gold nanoparticle hybrid effectively reduced the inflammatory response [200]. Consistently, reduced kynurenine production was shown to be associated with an increased inflammatory response in the lungs of aged individuals during an influenza infection [201]. Another study found kynurenine metabolism pathways were upregulated in the inflammatory disease, aortic atherosclerotic aneurysm, and the metabolism of kynurenines help downregulate inflammation [202]. With progression of the kynurenine metabolism pathways, tryptophan is ultimately broken down into small molecules such as NAD^+^, picolinic acid (PA), ATP, and CO_2_ [72], which each having different roles in the anti-inflammatory function of macrophages. NAD^+^ was found to limit inflammation and promote phagocytosis [203], whereas PA is an activator of macrophage pro-inflammatory function [204].

Tryptophan is transported by system L in macrophages [81]. M2-like macrophages expressing IDO suppress the immune response by promoting the function of Tregs, and inhibiting Teff cells via the KYN-AhR pathway [205, 206]. AhR activation in tumor-associated macrophages might not only depend on tryptophan metabolism within macrophages, but might also require *Lactobacillus* metabolized dietary tryptophan to indoles to drive TAMs to acquire an immunosuppressive phenotype [207]. This observation emphasizes the importance of amino acid metabolism of the microbiota in the tumor immune response. Furthermore, high activity of AhR in glioblastoma is associated with reduced overall survival, suggesting AhR might become a potential therapeutic target in some tumor settings [208].

#### Branched-chain amino acids (BCAAs)

BCAAs have both pro- and anti-inflammatory functions in macrophages [209]. In early stages of macrophage activation following lipopolysaccharide (LPS) stimulation, transamination by BCAT1 is increased instead of upregulating BCAAs uptake [210]. The inhibition of BCAT1 reduced itaconate production and limited flux through the tricarboxylic acid (TCA) cycle in macrophages, which is associated with reduced expression of immune responsive gene 1 (IRG1). While this contributes to the downregulation of macrophage pro-inflammatory function, a detailed mechanism remains unclear [210, 211]. *IRG1*, one of the highly upregulated genes in activated macrophages, functions to generate itaconic acid (also known as methylenesuccinic acid) through the decarboxylation of cis-aconitate, a TCA cycle intermediate [212]. Besides general analysis of BCAA metabolism, individual BCAA members such as leucine have also been studied as well. Supplementation of leucine decreases cholesterol levels by inhibiting cholesterol biosynthesis, and thus attenuates macrophage foam-cell formation, which is beneficial for preventing atherogenicity [213]. Thus, in lipid metabolism, the deprivation of leucine led to white adipose tissue (WAT) browning and lipolysis through GCN2, which helps to treat obesity [214]. However, there is currently no direct evidence showing BCAAs regulate tumor growth through their metabolism in macrophages.

#### Arginine

Generally, in macrophages, arginine is catabolized by inducible isoform of NO synthase (iNOS) and arginase. After stimulation of Toll-like receptors (TLRs), activated macrophages produce NO through iNOS to resist various pathogenic infections such as *Mycobacterium tuberculosis*, *Leishmania*, bacillus Calmétte-Guerin (BCG) [215-217]. For inflammatory disease, iNOS-dependent NO synthesis increases mesangial cell lysis leading to tissue fibrosis in the anti-thymocyte serum (ATS)-induced model of glomerulonephritis [218]. This suggests that the effects of iNOS-dependent NO synthesis favor progression towards inflammation. On the other hand, given NO serves as an important neurotransmitter, the production of NO by macrophages was shown to promote non-adrenergic, non-cholinergic inhibitory nerve stimulation [219]. Furthermore, arginase, was shown to be critical for *Leishmania* proliferation [220]. Arginase-1, an arginase subtype, regulates the status of macrophages to promote the resolution of arthritis [221]. Similarly, arginase-2, localized to mitochondria in macrophages, relieved inflammation by regulating IL-10 [222]. In addition, arginase promotes the accumulation of M2-like macrophages around arterial cell proliferation [223]. Given their similar metabolic substrates, iNOS and arginase also cooperated to control NO-mediated macrophage apoptosis [224].

Arginine undergoes different metabolic processing in M1-like and M2-like macrophages within the TME. In M1-like macrophages, arginine is metabolized to produce NO to inhibit tumor development [225, 226]. Whereas M2-like macrophages consumed arginine through arginase-1 to limit arginine accessibility to other anti-tumor immune cells such as Teff cells, which has a negative consequence on tumor killing [226]. Therefore, these results suggests that there may be a coordinated, or competition-based, mechanism between iNOS and arginase-1 for arginine [227]. Whether through classical or alternative activation mechanisms, the expression of SLC7A2 is induced in macrophages [228] which allows for uptake of arginine to a high level in activated macrophages [229]. SLC7A2 knockout mice show a decrease in uptake of arginine [228, 230]. Although the expression of iNOS and arginase-1 was not changed, the metabolism of arginine was reduced in SLC7A2-deficient macrophages [228]. In recent years, a third fate for arginine metabolism has been characterized in macrophages where arginine is metabolized by two enzymes, glycine amidinotransferase and guanidinoacetate methyltransferase, to generate creatine, which promotes M2-like macrophage polarization [231]. However, the maintenance of creatine concentrations in macrophages is through the transporter SLC6A8, which allows for the possibility to influence phenotypes of macrophages and modulate macrophage-mediated immune response though pharmacologically or genetically targeting SLC6A8 [231]. In breast cancer, supplementing sepiapterin, the precursor of tetrahydrobiopterin (BH4), which serves as a cofactor for NOS, redirects arginine metabolism in macrophages from the polyamines synthesis pathway to the NO pathway. This induces a shift of M2-like macrophages to M1-like macrophages and blocks STAT3-dependent expression of PD-L1 in tumor cells to suppress their growth [232].

#### Glutamine

Similar to arginine, glutamine also promotes M2 polarization, where deprivation of glutamine was shown to suppress M2-like macrophages and the production of the chemokine CCL22, potentially through the mTOR pathway [233]. Similarly, glutamine metabolites also play a role in M1/M2 polarization as well. The production of α-ketoglutarate via glutamine lysis promoted M2-like macrophage activation, and the low ratio of α-ketoglutarate/succinate enhanced pro-inflammatory functions of M1-like macrophages [234]. Furthermore, downregulation of α-ketoglutarate is controlled by isocitrate dehydrogenase (IDH), which is a downstream target of the nuclear receptor Nur77 (*Nr4a1*) [235]. Therefore, glutamine promotes anti-inflammatory functions of macrophages through Nur77-mediated transcription [236, 237]. Additional functions of glutamine in macrophages include promoting phagocytosis as metabolism of glutamine through glutaminase-1 is essential for macrophages to eliminate apoptotic cells [238]. In addition, supplementation of macrophages in culture promoted cell cycle progression [239]. However, other studies have suggested that dietary supplementation of glutamine reversed macrophage function in newborn mice [240], and glutamine might also suppress lysosomal function, anti-inflammatory phenotypes, and cell survival under certain conditions [241].

Glutamine deprivation in the TME may not serve as a pro-inflammatory factor, but rather may promote tumorigenesis. The consumption of glutamine in clear cell renal cell carcinoma led to a local glutamine deprivation such that macrophages secreted IL-23 due to HIF-1α induction to strengthen immunosuppressive function of Tregs in the TME [242].

### Dendritic cells

As the main antigen presenting cells (APCs), dendritic cells (DCs) are a key link between the innate immune function with the induction of adaptive immunity. DCs are the central regulator of adaptive immunity and immune tolerance, and their discoverer Ralph M. Steinman was awarded the 2011 Nobel Prize in Physiology or Medicine [243].

Dendritic cells are classified into various subtypes depending on their function or lineage differentiation. These subtypes include conventional dendritic cells (cDCs), plasmacytoid DCs (pDCs) and monocyte-derived DCs (moDCs). cDCs are primarily associated with T cell differentiation [244]. Moreover, in the TME, pDCs and moDCs are indispensable for tumor immunity [245, 246], and pDC have been proposed to serve as a biomarker in triple negative breast cancer (TNBC) and non-small-cell lung cancer (NSCLC) [247, 248]. Other studies suggest pDCs may contribute to attenuate tumor development [249, 250], although mechanisms for these observations have not been clarified. moDCs are indirectly associated to anti-tumor immune response through the promotion of T cell polarization towards Th1, Th2, and Th17 subtypes [251, 252]. However, DCs may function as a double-edged sword regarding tumor immunity. A role for DCs in promoting tumorigenesis are centered on the recognition and combination of immune checkpoints. DCs expressed CD80/CD86 (B7-1/B7-2) which bind to T cells expressing CTLA-4 to deliver co-stimulatory signals to suppress the T cell response [253]. Furthermore, DCs that highly expressed PD-L1 bind to PD-1 receptors of T cells to allow tumor cells to escape from elimination by cytotoxic T cells [254]. Given the widespread effects of DCs on immunity both inside and outside of the TME, it is important to discuss the roles of amino acids in regulating DCs (Fig. 3).

#### Tryptophan

IDO is typically considered an immune suppressive marker of DCs. In transplantation models in mice, both IDO^+^ DCs and tryptophan metabolites were efficient to reduce organ rejection, with enhanced effects observed when provided in combination [255]. This is likely due to IDO and its byproducts inhibiting Teff cell function which may be due to IDO attenuating the generation of memory CD8^+^ T cells and inhibiting the function of memory T cells in allograft rejection as well as proliferation [256]. Interestingly, the activity of IDO is regulated by Arg1, as the release of polyamines that are catabolized by Arg1 coerced DCs toward an immunosuppressive phenotype through the promotion of IDO1 phosphorylation [257]. This suggests the presence of amino acid metabolism crosstalk in DCs. Following catabolism by IDO, kynurenine was further catabolized by kynurenine 3-monooxygenase (KMO), and deficiency of KMO led to the accumulation of kynurenine thereby promoting the generation of Tregs to relieve colitis [258]. Kynurenine is converted to 3-hydroxykynurenine by KMO which is subsequently converted to 3-hydroxyanthranilic acid (3-HAA) by 2-amino-4-[3-hydroxyphenyl]-4-hydroxybutanoic acid (KYUN). While DCs influence Teff cells through regulating tryptophan metabolism, the metabolites of tryptophan including 3-HAA inhibited the activation status of DCs to suppress the T cell response after LPS stimulation [259]. In addition, upstream molecules target IDO to influence function of DCs as well. For instance, thymosin α1, a naturally occurring thymic peptide, induces IDO expression, and immunosuppressive function of DCs, by stimulating TLR and IFN signaling [260, 261]. Interestingly, the gut microbiota aids in tryptophan metabolism as well. Indole is produced from tryptophan by the gut microbiota, and is further metabolized to 3-indoxyl sulfate in the liver, which can contribute to regulating DCs anti-inflammatory functions [262]. Similar to biological molecules, inorganic molecules such as Zinc also influence DC maturation by reducing MHCII expression on the surface of DCs and promoting IDO catalysis, which restrains the proinflammatory response due to stimulation by TLR ligands [263]. In general, IDO is central to tryptophan metabolism in DCs and plays an important role in DCs immunosuppressive function.

The metabolism of tryptophan by IDO+ DCs may also activate Tregs and hence inhibit T cell-mediated anti-tumor immunity [264]. Interestingly, CTLA-4 expression in Tregs promotes IDO secretion by DCs, and the inhibition of CTLA-4 could effectively reduce the production of kynurenine and IFN-γ by DCs [265]. Such a mechanism between DCs and Tregs may exist as a positive feedback loop. In addition, kynurenine can serve as a signaling molecule to directly impede T cell anti-tumor response. Abnormal activation of protein kinases are a common pro-tumorigenic mechanism, and Bruton’s tyrosine kinase (BTK) is one such protein kinase that plays a crucial role in oncogenic signaling, especially in various B cell lymphomas [266]. In addition, BTK has been identified as a crucial regulator of the immunosuppressive function of myeloid cells including macrophages, myeloid-derived suppressor cells (MDSCs), and DCs [267, 268]. Initial studies indicated that activation of DCs could be inhibited by autocrine IL-10 secretion which activates BTK through the c-Src-PI3K-AKT-mTOR pathway [269, 270]. Subsequent studies indicated that the link between BTK and mTOR is mediated by amino acid signaling, and BTK may serve as an upstream regulator of the mTOR pathway. For instance, the BTK-IDO axis inhibits a GATOR2/GATOR1-mTORC1 derived tryptophan-sensitive differentiation pathway in DCs [271].

#### Arginine

The consumption of arginine by iNOS or arginase is also a feature of DCs inhibitory phenotype. As described above, polyamines, produced by arginase, promoted DCs to an IDO1-dependent immune suppressive phenotype [257]. When DCs were co-cultured with apoptotic cells, both the transcription of *iNOS* and the production of NO were increased due to the phosphorylation of STAT3, which converted DCs into suppressive DCs [272]. Similarly, following *Mycobacterium bovis* infection, genes associated with NO synthesis were upregulated, such as *SLC7A1*, *SLC7A2* and *iNOS*, which may contribute to bacterial survival [273]. The expression of arginase and iNOS are regulated by many factors. For instance, all-trans retinoic acid enhances arginase and iNOS expression, IL-35 induces arginase-1 expression in murine splenic DCs, and deficiency of complement 3 downregulates iNOS-2 expression which reduces Treg generation [273-276]. Moreover, when iNOS and arginase are inhibited by L-NAME and NOHA, respectively, CD80/CD86 ligand, which is important for T cell activation, is reduced on the membrane of DCs, which may contribute to pro-inflammatory functions of the immune system [277].

As for the TME, given DCs and macrophages share similarity in arginine metabolism, we speculate that arginine is also transported through SLC7A2 in DCs, but this needs further study. Arg1 is required for IDO induction by TGF-β in DCs, and TGF-β is necessary for the coexistence of Arg1 and IDO in DCs [257]. Thus, these results suggest that over expression of IDO and Arg1 in DCs leads to exhaustion of local tryptophan and arginine, which promotes immunosuppression and tumor progression.

### Myeloid-derived suppressor cells

Based on their origination as myeloid cells and immune suppressive capacity, inflammatory-associated cells that share similarities to neutrophils and monocytes in phenotype and morphology are defined as myeloid-derived suppressor cells (MDSCs) [278]. Based on phenotypic criteria, MDSCs are classified into granulocytic MDSC (G-MDSC) also named polymorphonuclear MDSCs (PMN-MDSC), and monocyte MDSC (M-MDSC). However, this current definition of MDSCs itself is ambiguous and uncertain, and studies have confirmed extensive heterogeneity of MDSCs cells [279, 280]. Furthermore, the existing definition of MDSCs is self-limiting and could be developed beyond this to associate with disease and pathological signals [281]. MDSCs are produced and accumulate in response to pathological situations such as cancer, bacterial infection, and trauma, and amino acids have been shown to play a role in their development and function (Fig. 3).

Arginine has been heavily studied in MDSCs, as MDSCs compete with Teff cells for arginine leading to dysregulation of arginine metabolism in T cells. In sepsis, MDSCs attenuate T cell function by metabolizing arginine to toxic molecules that impair T cell ζ chain expression [282]. In parasitic infection, MDSCs are induced by both larval and adult stages of *Heligmosomoides polygyrus bakeri* and increase their expression of arginase-1 and iNOS, which suggests that metabolism of arginine contribute to the maintenance of immunosuppressive function of MDSCs [283]. Another study observed that arginase-1 expressing MDSCs effectively inhibit the T cell response to alleviate tissue injury following hepatitis B viral infection [284]. Similarly, after physical injury, arginine availability is decreased and further induction of arginase-1 expression in MDSCs exhausts arginine levels to attenuate T cell activation and increase susceptibility to infection [111].

In mouse models of lung adenocarcinoma, Arg1 expression is elevated in G-MDSCs and both lung adenocarcinomas and squamous tumors [285]. Similarly, the number of Arg1 producing PMN-MDSCs increased in peripheral blood in renal carcinoma [286]. However, in human renal carcinoma Arg1 is released from PMN-MDSCs [286]. Besides Arg1, iNOS, is also an important enzyme for MDSC exercising their suppressive function. In MDSCs, Arg1 and NOS2 coordinately upregulate the cationic amino acid transporter 2 (CAT2, also named SLC7A2), which functions in the transport of extracellular arginine in mice [287]. Interestingly, another view has come into play for Arg1 in MDSCs where Arg1 is neither constitutively expressed in MDSCs nor necessary for MDSCs suppressive function, and induced expression of Arg1 in MDSCs does not influence MDSCs-mediated immune inhibition [288]. One possible explanation may be that samples in this study were isolated from bone marrow in B16 melanoma, MC38 colon carcinoma, or EL4 lymphoma mice, which differs from other studies, suggesting that the function of MDSCs may vary by organs, tissues, or cells, thus highlighting that the classification and definition of MDSCs may need further updating.

In a transplant model, MDSCs suppress macrophages-mediated cytotoxicity in an IDO-dependent manner, to overcome xenotransplantation organ rejection [289]. With regard to tryptophan metabolism in tumors, IDO also mediates immune suppressive effects of MDSCs in breast cancer and lung cancer [290, 291], and the upregulation of IDO in MDSCs depends on STAT3 phosphorylation [290]. Moreover, IDO associated with MDSCs also plays an important role in inducing the differentiation of Treg or Breg cells, which suppress anti-tumor immunity [292, 293]. Inhibiting glutamine metabolism also blocks expression of IDO in MDSCs and tumor cell growth [294]. Indicating there may exist a crosstalk between amino acids in controlling IDO expression. Interestingly, besides its role in immunosuppression, IDO expression in MDSCs contributed to tumor angiogenesis which depends on GCN2 [295].

There are counter viewpoints regarding glutamine metabolism in tumor associated MDSCs. For instance, inhibiting glutamine metabolism markedly suppressed the generation and mobilization of MDSCs, and further promoted the generation of anti-tumor inflammatory cells [294]. Whereas another study showed glutamine deprivation promotes the generation of MDSCs [296]. Both of these studies focused on 4T1 triple negative breast cancer, but the former was carried out in vivo, while the latter was primarily in vitro. This difference suggests that the function of MDSCs may be regulated by the microenvironment where other cell types or cytokines may provide signals to direct MDSC function. Moreover, there appears to be crosstalk between glutamine and arginine metabolism in MDSCs. In glutamine-limited medium, the activity of iNOS, but not Arg1, was up regulated, which suggests that iNOS is induced to enhance central carbon metabolism and a high bioenergetic status [297].

While amino acid metabolism may directly regulate MDSCs immunosuppressive function, key metabolic sensing molecules including mTOR and GCN2 are also involved in regulating MDSCs. In acute kidney injury (AKI), the mTOR inhibitor rapamycin protected mouse kidneys from AKI in vivo through the induction and recruitment of MDSCs to suppress an excessive immune response [298]. GCN2 was found to promote the translation of the transcription factor CREB2/ATF4 to promote MDSCs maturation in a melanoma mouse model, and deficiency of ATF4 and GCN2 reduced tumor growth [299]. However, while amino acid metabolism may play a role in tumor associated MDSCs, how amino acids affect MDSCs through the classical mTOR or GCN2 pathway requires further investigation.

Collectively, these results suggest that specific targeting of amino acid metabolic pathways in MDSCs may have therapeutic value.

## Therapeutic opportunities of targeting altered amino acid metabolism in immune cells

As discussed above, tumor cells as well as some immunosuppressive cells compete with anti-tumor immune cells for free amino acids. In addition, amino acids utilize several transporters in various cell types where they are detected by key sensors and metabolized by key enzymes. Thus, supplementing amino acids, or targeting transporters, converting enzymes, metabolites and/or sensors involved in amino acid metabolism are potential therapeutic strategies. Some of which are already undergoing clinical development (Tables 1 and 2).

**Table 1:** Clinical trials on amino acid supplementation

Type of amino acids	Intervention/treatment (design)	Type of cancer	NCT number	Phase	Status	Purpose
Glutamine	Dietary supplement of glutamine	Metastatic cancer	NCT04710290	III	Enrolling	Evaluate the efficacy of glutamine supplementation in patients with metastatic cancers undergoing chemotherapy
	Dietary supplement of glutamine	Head and neck cancer	NCT00006994	III	Terminated	Evaluate the efficacy and safety of L-Glutamine upon radiation therapy-induced oral mucositis in head and neck cancer patients
	Dietary supplement of standard-glutamine Dietary supplement of standard-glutamine-omega 3	Colorectal cancer	NCT01831310	IV	Completed	Evaluate the effect of postoperative glutamine-dipeptide supplemented parenteral nutrition on neutrophil functions and postoperative course of patients with colorectal cancer
	Drug: Glutamine	Colorectal Neoplasms	NCT01087658	III	Completed	Evaluate the efficacy of oral glutamine in the prevention of oxaliplatin-induced neurotoxicity in patients with colorectal cancer treated with oxaliplatin
	Drug: Glutamine	Kidney cancer	NCT00365768	II	Completed	Study the effects of glutamine in pediatric patients with cancer
	Dietary supplement of glutamine	Unspecified adult solid tumor	NCT00217724	Not Applicable	Terminated	Study how effect glutamine in preventing myalgia and/or arthralgia in patients who are receiving paclitaxel for cancer
	Drug: Glutamine	Advanced cancers	NCT01952847	III	Terminated	Study glutamine function in reliving sores, blisters, or inflammation in the mouth or esophagus
Arginine	Dietary supplement of arginine	Head and neck cancer	NCT00559156	II	Completed	Evaluate the impact of arginine for postoperative radio chemotherapy in patients with carcinoma of the head and neck
	Drug: oral L-arginine	Prostate cancer	NCT01105130	II	Completed	Study effect of L-arginine supplementation in treating erectile dysfunction and quality of life of prostate cancer survivors previously treated with radiation therapy

**Table 2:** Clinical trials on blocking amino acid metabolic pathways in combination with traditional therapeutics

Targets	Mechanism	Drugs	Combinatorial intervention	Type of cancer	NCT number	Phase	Status
IDO1	Inhibiting trp-kyn metabolism pathway and enhance anti-tumor immunity	Epacadostat	Biological: CRS-207	Platinum-resistant Ovarian Cancer, Fallopian Cancer, Fallopian Cancer	NCT02575807	I/II	Terminated
			Biological: DEC-205/NY-ESO-1 Fusion Protein CDX-1401 Drug: Poly ICLC	Fallopian Tube Carcinoma, Ovarian Carcinoma, Primary Peritoneal Carcinoma	NCT02166905	I/II	Completed
			Drug: Itacitinib	Solid Tumors	NCT02559492	I	Terminated
			Drug: Azacitidine Drug: Pembrolizumab Drug: INCB057643 Drug: INCB059872	Solid Tumors, Advanced Malignancies, Metastatic Cancer	NCT02959437	I/II	Terminated
			Biological: SV-BR-1-GM Biological: INCMGA00012 Drug: Low dose cyclophosphamide Biological: Interferon Inoculation	Breast Cancer Female, Breast Neoplasm Female	NCT03328026	I/II	Recruiting
			Drug: Nivolumab Drug: platinum chemotherapeutic drug	Lung Cancer	NCT03348904	III	Terminated
			Drug: Pembrolizumab Drug: Placebo	Urothelial Cancer	NCT03361865	III	Completed
			Drug: Pembrolizumab Drug: chemotherapeutic drug	Solid Tumor	NCT03085914	I/II	Completed
			Drug: Nivolumab Drug: Ipilimumab Drug: Lirilumab	Solid Tumor	NCT03347123	I/II	Terminated
			Drug: Retifanlimab	Endometrial Cancer	NCT04463771	II	Recruiting
			Drug: Pembrolizumab Biological: CRS-207 Drug: CY Biological: GVAX	Metastatic Pancreatic Adenocarcinoma	NCT03006302	II	Active, not recruiting
			Drug: Ipilimumab	Melanoma	NCT01604889	I/II	Terminated
			Drug: INCB001158 Drug: Pembrolizumab	Solid Tumors	NCT03361228	I/II	Terminated
			Drug: Pembrolizumab	Malignant Ovarian Clear Cell Tumor, Recurrent Ovarian Carcinoma	NCT03602586	II	Terminated
				Sarcoma	NCT03414229	II	Active, not recruiting
				Renal Cell Carcinoma (RCC)	NCT03260894	III	Active, not recruiting
				Head and Neck Cancer	NCT03358472	III	Active, not recruiting
				Urothelial Cancer	NCT03374488	III	Completed
		BMS-986205	Biological: Relatlimab Biological: Nivolumab	Advanced Cancer	NCT03459222	I/II	Recruiting
			Biological: Nivolumab Radiation: Radiation Therapy Drug: Temozolomide	Glioblastoma	NCT04047706	I	Recruiting
			Drug: Nivolumab	Endometrial Adenocarcinoma, Endometrial Carcinosarcoma	NCT04106414	II	Active, not recruiting
				Hepatocellular Carcinoma	NCT03695250	I/II	Active, not recruiting
		Indoximod	Radiation: Partial Radiation Radiation: Full-dose Radiation Drug: Temozolomide Drug: Cyclophosphamide Drug: Etoposide Drug: Lomustine	Glioblastoma, Medulloblastoma, Ependymoma, Diffuse Intrinsic Pontine Glioma	NCT04049669	II	Recruiting
			Drug: Ipilimumab Drug: Nivolumab Drug: Pembrolizumab	Melanoma	NCT02073123	I/II	Completed
		LY3381916	LY3300054	Solid Tumor, Non-Small Cell Lung Cancer, Renal Cell Carcinoma, Triple Negative Breast Cancer	NCT03343613	I	Terminated
		KHK2455	Avelumab	Urothelial Carcinoma	NCT03915405	I	Active, not recruiting
Arginase	Prevent arginine catabolize to immunosuppressive molecules and arginine exhaustion	INCB001158	Drug: chemotherapeutic drugs	Biliary Tract Cancer, Colorectal Cancer, Endometrial Cancer, Gastroesophageal Cancer, Ovarian Cancer, Solid Tumors	NCT03314935	I/II	Active, not recruiting
			Drug: Epacadostat (anti-IDO) Drug: Pembrolizumab	Solid Tumors	NCT03361228	I/II	Terminated
			Biological: Daratumumab SC	Relapsed or Refractory Multiple Myeloma	NCT03837509	I/II	Completed
AHR	Block the trp-kyn-AHR pathway	IK-175	Drug: nivolumab	Advanced Solid Tumors, Metastatic Solid Tumors, Urothelial Carcinoma	NCT04200963	I	Recruiting
				Head and Neck Squamous Cell Carcinoma	NCT05472506	I	Not yet recruiting
		BAY2416964	Drug: Pembrolizumab	Advanced Solid Tumors	NCT04999202	I	Recruiting
				Advanced Solid Tumors	NCT04069026	I	Recruiting

### Supplementation of amino acids

As summarized above, one reason why amino acid metabolism influences the function of anti-tumor immune cells is that several cell types including tumor cells will compete for free amino acids with anti-tumor immune cells. Thus, studies have been devoted to exploring the efficacy of free amino acid supplementation in cancer therapy (Table 1).

In cancer patients receiving concurrent chemoradiotherapy, dietary supplementation of arginine, glutamine and fish oil reduced the incidence of a series of postoperative complications, such as hematologic toxicities and mucocutaneous fistula [300, 301]. Arginine supplementation also showed efficacy when combined with chemotherapy drugs. For instance, combining arginine with docetaxel (DTX), an immunomodulatory chemotherapeutic agent, promoted anti-tumor phenotypes of DCs and reduced the proliferation of MDSCs in mouse models of breast cancer [302]. Supplementation of arginine increases infiltration of CD8^+^ T cells into colon carcinoma-bearing mice only when combined with of cyclophosphamide (CP) [303]. In addition, arginine induced a metabolic shift of activated T cells from glycolysis to oxidative phosphorylation to promote the generation of T cells with higher survival capacity and anti-tumor activity [137]. Consistent with these studies, arginine treatment markedly reduced the population of MDSCs and the expression of reactive oxygen species (ROS), and correspondingly enhanced the anti-tumor activity of effector T cells which was thought to be due to excessive consumption of arginine by MDSCs in the TME [304]. But in a clinical trial of colorectal cancer patients, arginine supplementation did not reduce MDSCs frequency. On the contrary, an arginine-treated cohort showed an increased frequency of polymorphonuclear MDSCs (PMN-MDSC) and monocyte MDSC (M-MDSC) compared to the placebo-treated cohort [305]. Although, when combined with other amino acids, some clinical trials show a positive benefit to cancer patients, and while arginine supplementation alone provided some effective anti-tumor efficacy when assessed in cell culture and animal models, mono therapy clinical trials of arginine supplementation have not been carried out.

Removal of tryptophan form the diet effectively inhibited tumor growth in animal models, and supplementing tryptophan or its catabolite indole-3-acetic acid rescued tumor growth and promoted an immunosuppressive tumor-associated macrophages phenotype [207]. Restriction of dietary serine impaired pathogen-driven expansion of T cells in vivo, but does not appear to influence overall immune homeostasis [143].

### Targeting SLC transporters

The expression of SLC transporters is regulated by many factors, and studies assessing the regulation of SLC amino acid transporters in immune cells suggests the possibility of a clinical transformation in cancer therapy. Here, we summarize the effect of gene therapy and pharmacological interventions on SLC family members (Table 3).

**Table 3:** Genetic or pharmacological inhibition of amino acid transporters on immune cells and the functional consequences

Immune cells	Gene	Aliases^b^	Substrates	Interventions	Biological functions	Ref (PMID)
T cells	*SLC1A5*	ASCT2	Asn	Genetic knockout in vitro	SLC1A5 is critical for uptake of Asn to promote CD8^+^ T cells activation	33,420,490
			Gln	Chemical inhibition by V-9302 in vitro	V-9302 suppresses tumor progression but does not affect CD8^+^ T cells	29,334,372
			Gln	Chemical inhibition by GPNA	Inhibition of SLC1A5 decreases TNF-α and IL-17 in PBMCs	30,541,099
	*SLC7A1*^a^	CAT1	Arg	Genetic knockout in vivo	Generation and persistence of memory T cells can be reprogrammed by SLC7A1 transporting Arg, partly via mTORC1	33,636,132
	*SLC7A5*^a^	LAT1	Met, Leu, Ile, Val, Phe, Trp	Genetic knockout in vitro	SLC7A5 is required for activated T cells to sustain Myc protein levels	32,022,686
			Leu	Genetic knockout in vivo	TCR controls *SLC7A5* expression is critical for metabolic reprogramming in T cells	23,525,088
			Met	Genetic knockout in vivo	*SLC7A5* null CD4^+^ T cells show a decrease of methyl donors	30,916,644
			Kyn	Genetic knockout in vivo	Kyn transport in T cells is controlled and mediated through SLC7A5	29,773,791
	*SLC7A11*^a^	XCT	not mentioned	Genetic knockdown in vivo; Chemical inhibition by SAS in vitro;	Proliferation and function of Tregs was inhibited by depleting *SLC7A11*, but restored by DMF-induced upregulation of SLC7A11	34,004,141
	*SLC38A1*	SNAT1	Gln	Genetic overexpression in vitro	Overexpression of *SLC38A1* enhance mitochondrial function of CD4^+^ T cells	30,305,738
			Ala	Genetic knockdown in vitro	Uptake of exogenous alanine by SLC38A1 is critical for primary CD4^+^ T cell mitogenesis	31,533,027
	*SLC38A2*	SNAT2	Gln	Genetic knockout in vivo	Generation and persistence of memory T cells can be reprogrammed by SLC38A2 transporting Gln, partly via mTORC1	33,636,132
	*SLC43A2*	LAT4	Met	Genetic knockdown in vitro	The downregulation of *SLC43A2* increase apoptosis of Tregs due to the decrease in Met uptake	36,260,753
B cells	*SLC7A5*^a^	LAT1	Leu	Expression stimulated by CpG ODN; blocked by BCH in vitro	Leu transport trough SLC7A5 is critical for B cell immune function	30,092,695
	*SLC15A4*		His	Genetic knockout in vivo	SLC15A4 is required for B cell mTOR-dependent IFN-I response to TLR7 stimulation, contributing to lysosome environment optimization and autoantibody production	25,238,095
NK cells	*SLC1A5, SLC7A5*^a^	ASCT2, LAT1	Gln	Targeted block by GPNA (SLC1A5) and D-phenylalanine (SLC7A5) in vitro	IL-2 priming increases SLC1A5 and SLC7A5 expression to promote IFN-γ production in NK cells	28,784,848
	*SLC3A2/SLC7A5*^a^	CD98/LAT1	Gln, Leu	Expression stimulated by IL-18 in vitro	IL-18 stimulates overexpression of SLC3a2/SLC7A5, inducing metabolic changes in NK cells	30,696,773
	*SLC7A5*^a^	LAT1	Gln, Leu	Genetic knockout in vivo	Leu is required for mTORC1 signaling and Gln is required to sustain cMyc expression when transported by SLC7A5 in NK cells	29,904,050
Macrophages	*SLC1A2, SLC1A3*	EAAT2, EAAT1	Glu	Competitively inhibited by D-Asp in vitro	Inhibition of SLC1A2 and SLC1A3 increase the glutamate-induced GSH in macrophages derived from monocytes	11,698,255
	*SLC7A2*^a^	CAT2	Arg	Genetic knockout in vivo	*SLC7A2* KO reduces Arg uptake in macrophages, and impairs the catabolism of Arg	16,670,299
DCs	*SLC3A2/SLC7A11*^a^	CD98/XCT	Glu, Cys	Targeted block by L-homocysteic acid in vivo	The block of SLC3A2/SLC7A11 heteromeric partners in DCs disrupts GSH homeostasis and may impair immunity in the diseased host	20,733,204
	*SLC15A4*		His	Genetic knockout in vivo	*SLC15A4* mutation leads to defective endosomal TLR signaling might due to compromised transport of His from endlysosome to cytosol in pDCs	35,349,343
			His	Genetic knockout in vivo	*SLC15A4*-deficient DCs show reduced caspase-1 cleavage and IL-1β production by modulating mTORC1 function	36,031,853
MDSCs	*SLC7A2*^a^	CAT2	Arg	Genetic knockout in vivo	Loss of *SLC7A2* impairs the suppressive function of MDSCs in vitro, leads to increase in T cell numbers and decrease of tumor growth in vivo	26,491,198

*Ala* Alanine; *Arg* Arginine; *Asn* Asparagine; *Asp* Asparticacid; *BCH* 2-amino-2-norbornanecarboxylic acid; *DMF* Dimethyl fumarate; *Gln* Glutamine; *Glu* Glutamate; *GpG ODN* CpG oligonucleotide; *GPNA* L-g-glutamyl-p-nitroanilide; *GSH* Glutathione; *His* Histidine; *IFN* Interferon; *Ile* Isoleucine; *Kyn* Kynurenine; *Leu* Leucine; *Met* Methionine; *mTORC1* Mechanistic target of rapamycin complex 1; *PBMCs* Peripheral blood mononuclear cells; *pDCs* plasmacytoid dendritic cells; *Phe* Phenylalanine; *SAS* Sulfasalazine; *SLC* Solute carrier; *TCR* T-cell receptor; *TLR* Toll-like receptor; *TNF* Tumor necrosis factor; *Trp* Tryptophan; *Val* Valine

^a^Heteromeric amino acid transporters (HATs) comprise a group of SLC membrane proteins: a light chain subunit from an SLC7 family member and a heavy chain subunit from the SLC3 family

^b^Aliases of these SLC members only mention the most known in amino acids metabolism

Besides direct regulation of SLC genes or proteins, recent studies also provide a series of potent targets to design effective and specific therapeutics. *c-Myc*, a well-known oncogene, controls the transcription of SLC transporters, but which transporters are regulated in T cells and tumor cells differ [306, 307]. Interestingly, amino acids transported by SLC transporters activate mTORC1 to increase *c-Myc* expression [308, 309], thus there may exist a positive feedback loop among *c-Myc*, SLC transporters, and mTORC1. In addition, mTORC1 regulates the transcription factor ATF4 to balance cellular amino acid supply with demand for protein synthesis through regulating SLC transporters, and loss of ATF4 reduces amino acid uptake [310]. Similarly, *c-Myc* was also found to regulate SLC1A5 expression in B cells, which was curbed by the microRNAs let-7adf [147]. Similarly, another microRNA, miR-31-5p, suppressed the expression of SLC15A4 in pDCs and downregulates IFN-I production, but the mechanism remains unclear [311]. Under hypoxic conditions, SLC38A2 was upregulated in a HIF-1α-dependent manner [312] whereas SLC7A5 is upregulated by HIF-2α [313]. Other molecules such as interleukins induce SLC transporter activity as well. IL-2, IL-15 and/or IL-18 upregulated a series of SLC transporters in effector T cells and NK cells [32, 184-186, 314]. Moreover, post-translational modifications, such as phosphorylation, N-glycosylation, acetylation, palmitoylation, ubiquitination and SUMOylation, also play important roles in SLC transporter functions as well [315].

In view of these processes that affect SLC transporter activity, overexpression of certain transcription factors, cytokines, and chemokines may upregulate SLC transporters in immune cells to enhance amino acid uptake from the TME. The development of chimeric antigen receptor (CAR) immune cells may allow for exploitation of these pathways to promote anti-cancer effects. For instance, CAR-NK cells increased their function and survival through autocrine signaling of IL-15 [316]. While these regulatory mechanisms warrant more intensive study, elevating expression or activity of amino acid transporters may further empower these CAR-NK or CAR-T cells.

On the other hand, identifying mechanisms to downregulate SLC transporter activity in pro-tumor immune cells may also makes sense. Existing SLC transporter inhibitors mostly focused on tumor cells, and targeting SLC1A5, SLC3A2, or SLC7A5 has shown anti-tumor efficacy [317–319]. Therefore, some immune cells such as Tregs or MDSCs which suppress the immune response may also serve as effective targets of SLC transporter inhibitors.

### Targeting amino acid metabolism enzymes

IDO and TDO are key enzymes involved in tryptophan metabolism, and exist in tumor cells and immune cells except lymphoid cells. Both catabolize tryptophan to kynurenine which suppresses anti-tumor effects of lymphoid cells. Therefore, studies have focused on developing IDO or TDO inhibitors to improve the anti-tumor effects of immune cells, and IDO1, IDO2 and TDO inhibitors have already been summarized [320]. However, IDO1 inhibitor monotherapy did not show clinical efficacy [321], which may be due to the fact that tryptophan can be catabolized by IDO2/TDO as well, and thus the combined therapy of inhibitors of these three enzymes may show increased responses. Recent efforts to develop dual inhibitors of IDO and TDO have elicited encouraging results with respect to anti-tumor effects [322–326]. Moreover, IDO inhibition shows synergistic effects on tumor cytotoxicity with immune checkpoint inhibitors (Table 2), such as PD-1/PD-L1 blockade and CTLA-4 blockade [327–329], and similar synergistic effects were also reported for chemotherapy [330-332] and radiotherapy [333]. Consistent with this notion, supplementation with tryptophan or IDO inhibitors enhanced CD8^+^ T cells to induce apoptosis of co-cultured cancer cells and increase the infiltration of CD8^+^ T cells into cancer nests. Furthermore, supplementation of tryptophan may help IDO inhibition and PD-1 blockade in anti-cancer treatments [334].

Pharmacologic inhibition of the BTK-IDO axis and deletion of BTK/IDO genes led to the robust differentiation of inflammatory DCs and promoted anti-tumor T cell responses both in vitro and in vivo [271]. With respect to MDSCs, Ibrutinib, an irreversible inhibitor of BTK and IL2-inducible T-cell kinase (ITK), suppressed MDSC generation and downregulated mRNA expression of IDO in vitro [268]. From these studies, one can speculate that BTK-IDO may be a universal signaling pathway promoting immunosuppressive responses in various cell types, which may be a potential target of tumor therapy. Overall, studies with inhibitors of the IDO/TDO pathway display an inspiring prospect of clinical translation but may need to find additional possibilities for combination therapy in the future.

Metabolism of kynurenine by kynurenine-3-monooxygenase (KMO) led to dysfunction of pDCs and immunosuppressive activity in multiple myeloma. As a result, inhibition of KMO triggered both specific T cell and NK cell cytotoxic activity [335]. Interestingly, there was also a synergistic effect when KMO inhibition was combined with PD-L1 blockade. Besides being converted into immunosuppressive metabolites, kynurenine directly inhibits effector T cell anti-tumor functions which is rescued by tetrahydrobiopterin (BH4) [336]. Furthermore, attempts to identify mechanisms to eliminate kynurenine and/or other immunosuppressive metabolites in the TME may become an effective way to inhibit tumor development. PEGylated kynureninase, also referred to as PEG-KYNase, degraded kyn into non-immunosuppressive metabolites, which increased proliferation of infiltrating CD8^+^ T cells [337].

Arg1 is another key factor of immunosuppressive myeloid cells and tumor cells. A small-molecule inhibitor of Arg1, CB-1158, resulted in a pro-inflammatory shift within the TME, and effectively reduced tumor growth [338]. OAT-1746, another Arg1 inhibitor, completely abrogated the immunosuppressive function of Arg1 in extracellular vesicles secreted from Arg1^+^ cells [339]. The novel Arg1/2 inhibitor, compound 9, restored effector T cell function but did not show anti-tumor effects in in vitro experiments [285]. A recent study revealed a novel synthesized inhibitor effectively increased arginine concentrations in the serum [340], but whether it could hinder tumor growth has not been investigated. Interestingly, a traditional Chinese medicine Shaposhnikov root extract Prim-O-glucosylcimifugin (POG) effectively inhibited the immunosuppressive function of PMN-MDSCs and exhibited a synergetic anti-tumor effect with PD-1 blockade by inhibiting arginine metabolism in PMN-MDSCs [341]. Similarily, iNOS inhibitors promote an anti-tumor immune response. In cutaneous squamous cell carcinoma, MDSCs impair vascular E-selectin expression through iNOS-induced NO production, and N(ω)-nitro-L-arginine(L-NNA), an iNOS inhibitor, restored vascular E-selectin expression to enhance T cell recruitment [342]. Both the Arg1 inhibitor NOHA (NG-Hydroxy-L-arginine) and iNOS inhibitor L-NAME (N^ω^-nitro-L-arginine methyl ester) upregulate expression of co-stimulatory molecules in DCs, facilitating the antigen presentation function of DCs [277].

Dihydrolipoamide succinyl transferase (DLST), a mitochondrial enzyme and subunit of the α-KGDC complex within the TCA cycle, was found to associate with ovarian cancer. Deprivation of glutamine reduced DLST expression in MDSCs and further rescued anti-tumor immune function [343].

### Target amino acids sensors

As a kyn sensor, AhR plays a critical role in the try-IDO/TDO-kyn-AhR pathway. Thus, it is generally recognized as a therapeutic target, both in immune cells and tumor cells. This immune suppressive axis could be an effective target by selective blockade of AhR to delay the progression of IDO/TDO overexpressing tumors and enhance anti-tumor efficacy when combined with immune checkpoint blockade. To this end, we have summarized clinical trials using AhR inhibitors (Table 2). 3′,4′-dimethoxyflavone (DMF) was reported to be an effective AhR inhibitor that blocks the formation of nuclear AhR complexes in TCDD induced breast cancer cells [344]. DMF markedly enhanced PD-1 blockade in CD8^+^ T cells when combined with carboxyamidotriazole [345]. Consistently, AhRil (CH-223191), another AhR inhibitor, promotes pro-inflammatory polarization of macrophages and increases infiltration of CD8^+^ T cells, leading to improved immune checkpoint blockade therapy with PD-1 antibody [95, 207, 346].

GCN2 senses amino acid deficiency, and negatively regulates the anti-tumor capacity of T cells. Disruption of GCN2 in T cells prevents inhibition of their proliferation induced by IDO^+^ DC cells both in vivo and in vitro [85], but it has yet to be associated with GCN2 amino acid sensing capability. Subsequent studies of GCN2 inhibition mainly focused on tumor cells [347–349]. Although a role for GCN2 in amino acid metabolism and the anti-tumor efficacy of GCN2 inhibition has been demonstrated, whether GCN2 inhibition is a potential therapeutic strategy to target tumor-associated T cells needs further evaluation.

The metabotropic glutamate receptor 2/3 (mGluR2/3) is expressed in MDSCs, and the mGluR2/3 antagonist LY341495 reduced the immune inhibitory function of MDSCs and further attenuated B16 melanoma growth [350].

## Conclusions and future perspectives

With the application of ultra-sensitive metabolic detection technologies, metabolic reprogramming of amino acids has been extensively identified in various types of malignancies. These studies have demonstrated that amino acid metabolism is an essential layer of regulation on immune cells to impact human tumorigenesis. As central amino acid sensors, mTOR and GCN2 integrate nutrient availability signals to regulate growth, polarization, and functions of cancer-associated immune cells. Amino acid uptake by immune cells is facilitated jointly by multiple amino acid transporters in the SLC family, whose functions are still not fully understood and deserves further comprehensive studies. Moreover, a series of rate-limiting enzymes control amino acid metabolism to regulate immune cell function in the TME.

However, only limited studies have shed light on the mechanisms by which amino acids influence tumorigenesis through SLCs, key enzymes involved in catalysis, and targeted sensors, to influence immune cell phenotypes. Most studies have focused on the well-defined amino acid regulatory pathways in immune cells, such as the uptake process and the function of metabolites, lacking a detailed understanding of mechanisms that drive metabolic reprogramming of immune cells. Moreover, there is increasing knowledge of T cell metabolism, but metabolic status of other infiltrating immune cells such as neutrophils or eosinophils have assessed. Similarly, with respect to amino acids, most studies have focused on a small subset of amino acids. The roles of many other amino acids in tumor-associated immune cells, as well as their metabolic processes and functional metabolites, require further investigation. In addition, dynamic regulation of metabolism has not been taken into consideration in most scenarios since cancer development and anti-tumor immunity is a spatiotemporally heterogenic process.

Therapeutic potential of targeting aberrant amino acid metabolism has been recently evaluated to enhance anti-cancer immunity. Compared to monotherapy (anti-PD-1 or chemotherapy), combination therapy with either amino acid supplementation or metabolic enzyme inhibitors enhances efficacy. Moreover, manipulation of amino acid metabolism may also improve immunotherapy with CAR-T cells. However, one should bear in mind that amino acids are essential metabolites required for numerous physiological processes and thus caution should be taken to avoid systemic cytotoxicity by targeting these metabolic pathways for cancer therapy. In this regard, novel delivery systems should be applied to modulate amino acid levels in specific immune cells within the TME.

Due to the complex architecture of the TME, developing and using new cell culture system such as organoids, will provide a more physiologically relevant environment to model the TME in vitro. By adding infiltrating immune cells into organoid cultures will provide a platform to quantitatively study metabolic interaction between various immune cells and tumor cells. In addition, the ability to supplement amino acids and metabolites into organoid cultures will be a valuable method to evaluate whether these molecules are necessary and sufficient for anti-tumor immunity. These techniques will promote an in-depth understanding of the reprogramming of amino acid metabolism in tumor-associated immune cells.

**Fig. 4:**
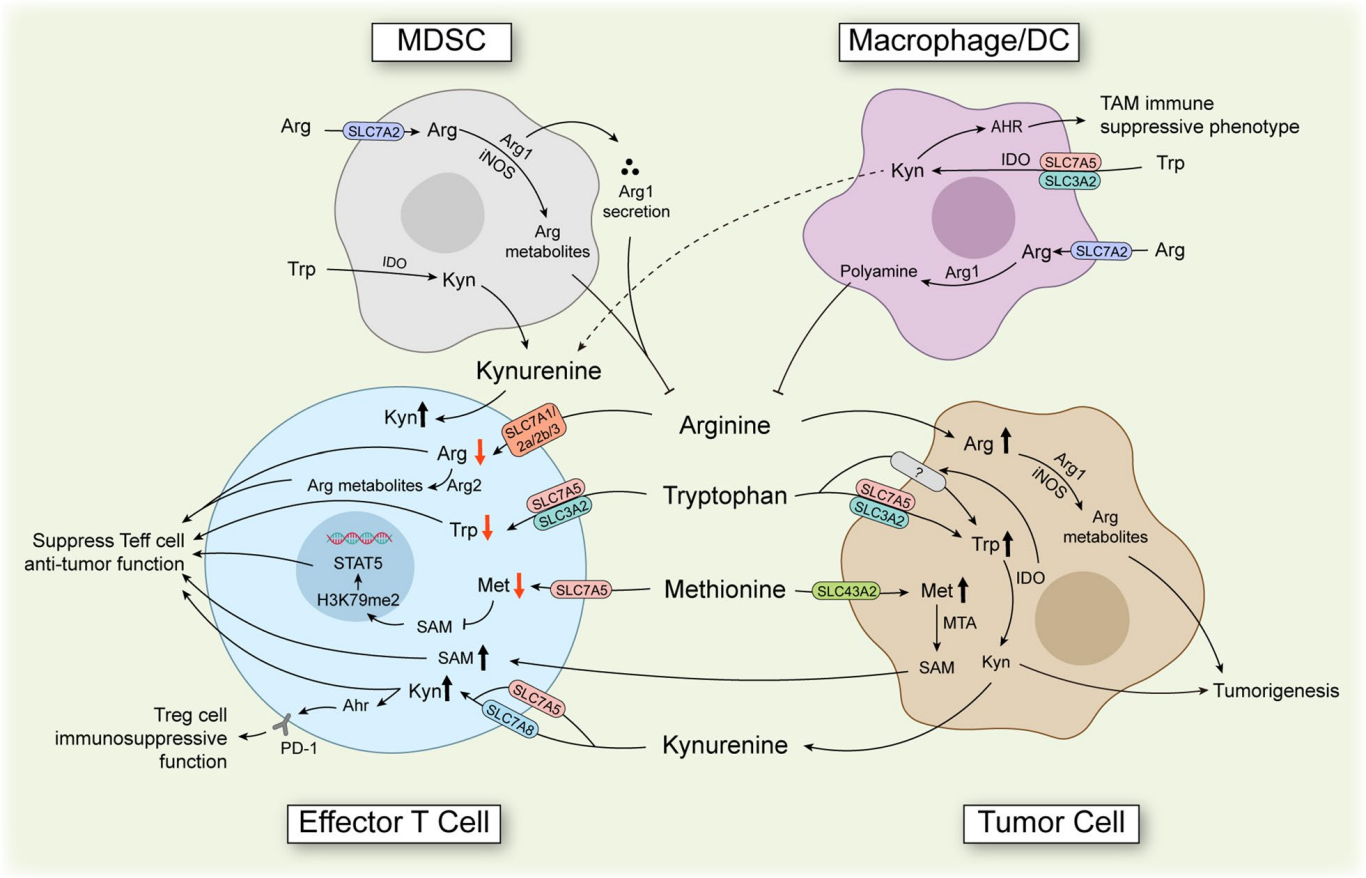
Amino acid competition in the TME. T cells compete with macrophages, MDSCs, and tumor cells for several amino acids, which influence the anti-tumor functions of T cells. Moreover, these cells produce kynurenine to suppress T cells, emphasizing the complexity of amino acid metabolism in the TME. The dashed line represents two lines that do not intersect. The arrow line represents activation and the line with a bar at the end represents inhibition. *AHR* Aryl hydrocarbon receptor; *Arg* Arginine; *Arg1/2* Arginase-1/2; *DC* Dendritic cell; *IDO* Indoleamine 2,3-dioxygenase; *iNOS* Inducible isoform of NO synthase; *Kyn* Kynurenine; *MDSC* Myeloid-derived suppressor cell; *Met* Methionine; *MTA* Methylthioadenosine; *PD-1* Programmed death 1; *SAM* S-adenosylmethionine; *STAT5* Signal transducer and activator of transcription 5; *TAM* Tumor-associated macrophages; *Trp* Tryptophan

Together, tremendous progress has been made in this field, but there are still many outstanding questions in reprogramming of amino acid metabolism in the TME. Moreover, amino acid competition between immune cells and cancer cells appears to be a widely observed phenomenon (Fig. 4). However, the underlying regulatory mechanisms are just beginning to be explored. While there will no doubt be broadly functioning molecular mechanisms, there will also likely be context-dependent factors driving amino acid competition in different cancer types. Further mechanistic insights into this fundamental nutrient metabolism rewiring will identify novel therapeutic targets to restore anti-tumor immunity in the TME and improve immunotherapeutic efficiency in the clinic.

## Data Availability

Not applicable.
